# Synergetic Relay Between Atomic Hydrogen and Chlorine Radicals Enables Efficient Nitrate‐to‐Nitrogen Gas Conversion

**DOI:** 10.1002/advs.202522147

**Published:** 2025-12-23

**Authors:** Yongjie Wang, Ying Tao, Chi Zhang, Shan Hu, Yuxin Shi, Jixing Wang, Jiacheng Zhang, Chen Cheng, Ying Wang, Guisheng Li, Zichao Lian, Dieqing Zhang

**Affiliations:** ^1^ The Education Ministry Key Lab of Resource Chemistry Shanghai Key Laboratory of Rare Earth Functional Materials and Shanghai Frontiers Science Center of Biomimetic Catalysis Joint International Research Laboratory of Resource Chemistry of Ministry of Education Shanghai Normal University Shanghai China; ^2^ School of Materials and Chemistry University of Shanghai for Science and Technology Shanghai China; ^3^ State Key Laboratory of Water Pollution Control and Green Resource Recycling Shanghai Institute of Pollution Control and Ecological Security College of Environmental Science and Engineering Tongji University Shanghai China

**Keywords:** nitrate removal, nitrogen gas selectivity, reactive hydrogen and chlorine radicals, tandem radical‐relay reaction, wastewater treatment

## Abstract

The excessive discharge of nitrate (NO_3_^−^) contamination in wastewater can lead to eutrophication of aquatic ecosystems, calling for technologies that can selectively reduce it to non‐toxic and harmless nitrogen gas (N_2_) reducing the secondary pollution risks. This work, for the first time, reports a synergic reduction−oxidation process that achieves highly efficient conversion of NO_3_^−^ to N_2_ via a relay strategy using reactive atomic hydrogen (H*) and chlorine radicals (Cl•). The photoelectrocatalytic system comprising a CuO‐Fe_3_O_4_/nickel foam (CuO‐Fe_3_O_4_/NF) and TiO_2_ nanotube arrays as the cathode and photocathodes, respectively, achieving a NO_3_^−^ removal rate of 97.4% and N_2_ selectivity close to 100%, while significantly suppressing the key bridging intermediate NH_4_^+^ accumulation and outperforming most reported values up to date. Mechanistic studies reveal that the cathodic CuO‐Fe_3_O_4_ component achieves the strong adsorption ability at CuO sites and hydrogenation reaction at Fe_3_O_4_ sites for activating H_2_O to generate reductive atomic H*, then highly selectively generate the NH_4_^+^ by spatial decoupling adsorption‐transformation processes. Subsequently, in‐situ generated Cl• by the photoanode TiO_2_, effectively scavenges and oxidizes NH_4_^+^, ultimately converting it to N_2_ via the tandem radical‐mediated reactions. Our discovery provides a sustainable strategy and drive great advances for removing nitrate pollutants in real aquatic environments.

## Graphical abstract

Using photoelectrochemical (PEC) deep denitrification technology, a novel synergistic reduction‐oxidation process enables efficient conversion of nitrate (NO_3_^−^) to nitrogen (N_2_), while suppressing key intermediate ammonium (NH_4_^+^) and toxic nitrite (NO_2_^−^) accumulation. This reductive H*‐oxidative Cl• coupling forms an efficient “radical‐relay” chain, achieving 97.4% NO_3_^−^ removal and near‐100% N_2_ selectivity.

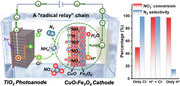

## 1 Introduction

The growing concern over nitrate (NO_3_^−^) contamination represents a widespread global environmental issue, primarily arising from agricultural fertilization, industrial effluents, and domestic wastewater [1]. Elevated nitrate levels can lead to eutrophication in aquatic ecosystems and human health [2]. More importantly, when ingested through drinking water, nitrate can be reduced to nitrite (NO_2_^−^), which is linked to methemoglobinemia (also known as “blue baby syndrome”) and the formation of carcinogenic N‐nitroso compounds [3, 4]. As a result, stringent regulations have been implemented worldwide limiting nitrate concentrations in drinking water—for instance, the World Health Organization recommends a guideline value of below 50 mg L^−1^ as NO_3_^−^ [5]. These concerns highlight a critical need for efficient, cost‐effective, and environmentally sustainable technologies for nitrate removal.

Currently, various strategies have been adopted for nitrate removal, including biological nitrification‐denitrification and chemical catalytic reduction methods [6, 7, 8]. However, the biological technology in efficient water treatment is constrained by slow reaction kinetics, sensitivity to water quality (e.g., dissolved oxygen), and the potential for residual biomass and harmful intermediates (e.g., NO_2_^−^, N_2_O) [9, 10]. Chemical catalytic hydrogenation reduction utilizes hydrogen (H_2_) to produce the reactive atomic hydrogen (H*) as a clean reductant over noble metal catalysts (e.g., Pd, Ru) for selectively converting NO_3_^−^/NO_2_^−^ into harmless nitrogen gas (N_2_) [11, 12, 13], which is hindered by the low solubility of H_2_ in water resulting into the mass transfer limitations to hamper reaction kinetics and the slower reduction rate of the critical intermediate, nitrite (NO_2_^−^), than that of nitrate, leading to its undesirable accumulation. These approaches often fail to strike a balance between removal efficiency and cost‐effectiveness [14].

Photoelectrocatalytic (PEC) technology is regarded as a promising solution to degrading pollutants due to its environmental friendliness, and the ability to utilize renewable sunlight to generate the electric energy [15, 16, 17, 18, 19, 20]. The ideal product of nitrate reduction is high‐value ammonia or non‐toxic nitrogen. For example, Mi et al. treated nitrite to ammonia‐N by using Co and Ni catalysts on GaN/Si photoelectrodes governed by H*‐mediated reduction and electron transfer processes. The reductive atomic H* poses high reduction ability to efficient removal of nitrite [21]. However, the lack of strong NO_3_^−^ absorption limited the conversion efficiency at the anode electrode. In addition, Zhou et al. developed a PEC system via the selective oxidation removal of NH_4_^+^ to N_2_ using the chlorine (Cl•) radicals generated by the reaction of chloride ions (Cl^−^) and photoinduced hole (h^+^) at the WO_3_ photoelectrode [22]. But, the restriction of the NO_3_^−^ reduction to NH_3_ at the cathode electrode occurred. Therefore, achieving the dual processes in one system with high selectivity is also a big challenge due to suitable matching degree of the two electrodes. Furthermore, although atomic H* and Cl• radicals have been extensively studied individually in reductive and oxidative processes via one‐directional conversion of inorganic nitrogen, respectively, their synergistic integration into a continuous reaction system for the deep removal of nitrate with precise steering toward N_2_ formation remains an unexplored frontier.

**SCHEME 1:**
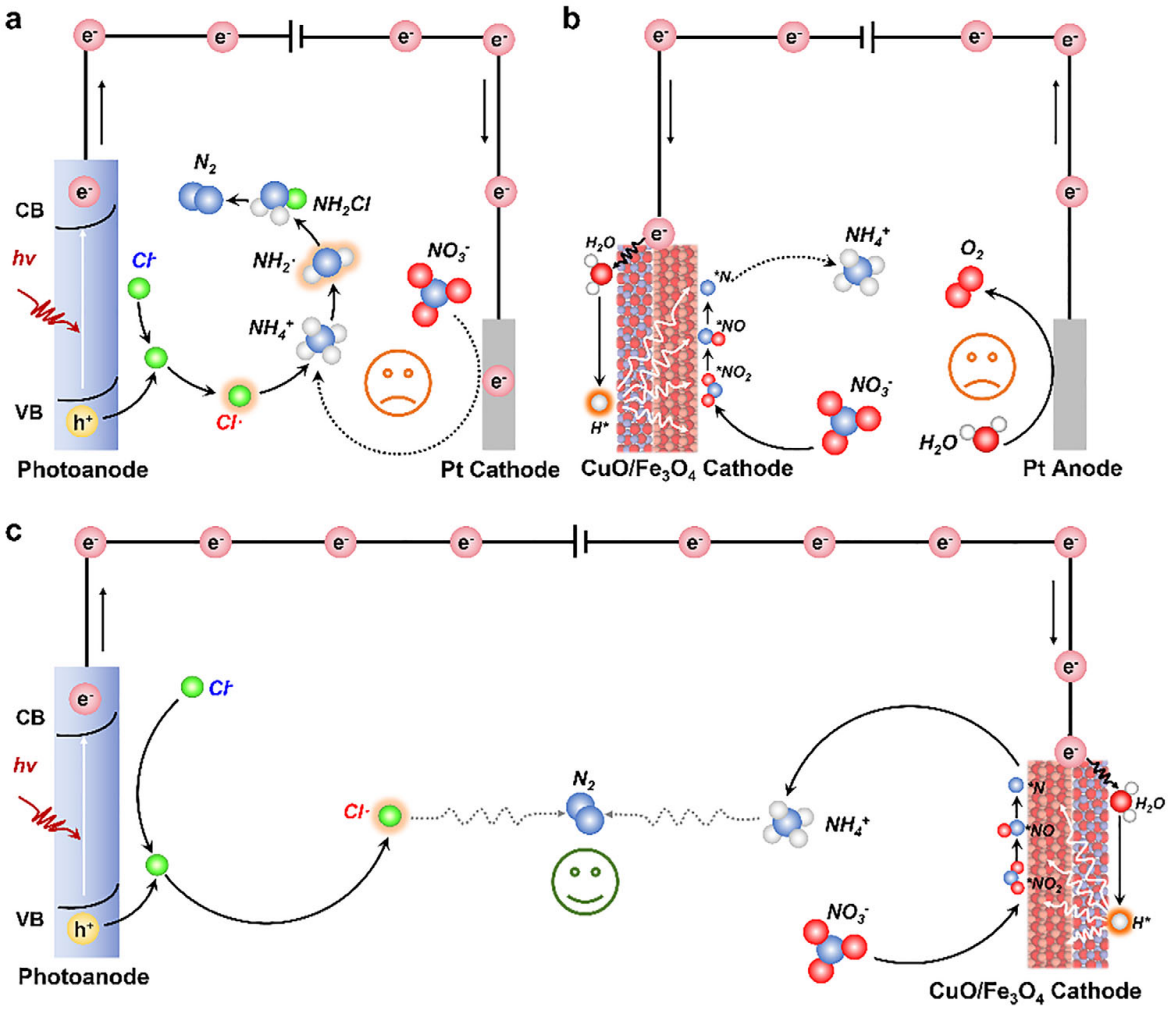
(a) NO_3_^−^ electroreduction to NH_4_^+^ and then oxidation to N_2_ by only oxidative chlorine radicals (Cl•) in the PEC system. (b) NO_3_^−^ electroreduction to NH_4_^+^ by only reductive atomic hydrogen (H*) in the EC system. (c) Proposed denitrification mechanism of the radical‐relay PEC system. Efficient removal of the NO_3_^−^ contaminants to N_2_, significantly suppressing the key bridging intermediate NH_4_^+^ accumulation was achieved by a novel synergic reduction−oxidation process using the PEC deep denitrification technology, where the coupling reductive H* and oxidative Cl• construct an efficient “radical‐relay” chain for wastewater treatment.

Inspired by the above analysis, we propose a novel synergic reduction−oxidation process using the PEC deep denitrification technology for efficient removal of the NO_3_^−^ to N_2_, significantly suppressing the key bridging intermediate NH_4_^+^ accumulation, where the ingenuity of this strategy lies in the clever coupling of two highly reactive radicals: reductive atomic hydrogen (H*) and oxidative chlorine radicals (Cl•) to construct an efficient “radical‐relay” chain in the PEC system, compared with only Cl• radical in PEC system and only H* radical in the PEC system, as shown in Scheme 1. Specifically, the cathodic CuO‐Fe_3_O_4_/Ni foam (CuO‐Fe_3_O_4_/NF) can realize high‐selectivity reduction of NO_3_^−^ to NH_4_^+^ by the reductive H* and simultaneously, the generated NH_4_^+^ reacts with Cl• in‐situ activation of chloride ions (Cl^−^) produced by a 1D nanotube array TiO_2_ (TiO_2_‐NTAs) photoanode under solar irradiation, achieving nearly 100% N_2_ selectivity and a 97.4% nitrate removal rate, outperforming most reported values up to date. Furthermore, this radical‐relay system exhibits exceptional stability, with no significant decline in NO_3_^−^‐to‐N_2_ removal efficiency and selectivity observed after 40 cycles, also displaying excellent nitrate removal performance in natural real lake. In‐situ experimental and theoretical results reveal that CuO facilitates the rate‐determining step of nitrate reduction (NO_3_^−^ to NO_2_^−^), while Fe_3_O_4_ catalyzes water splitting to generate H* for accelerating the intermediates to ammonia, which is subsequently converted with high selectivity to N_2_ by oxidative Cl• generated at the photoanode. The new paradigm of “reduction‐oxidation synergy” and “radical‐relay” revealed herein not only provides a transformative technological strategy for nitrate pollution control but also holds significant theoretical importance for understanding complex reaction networks in multi‐radical coupled systems.

## 2 Results and Discussion

**FIGURE 1:**
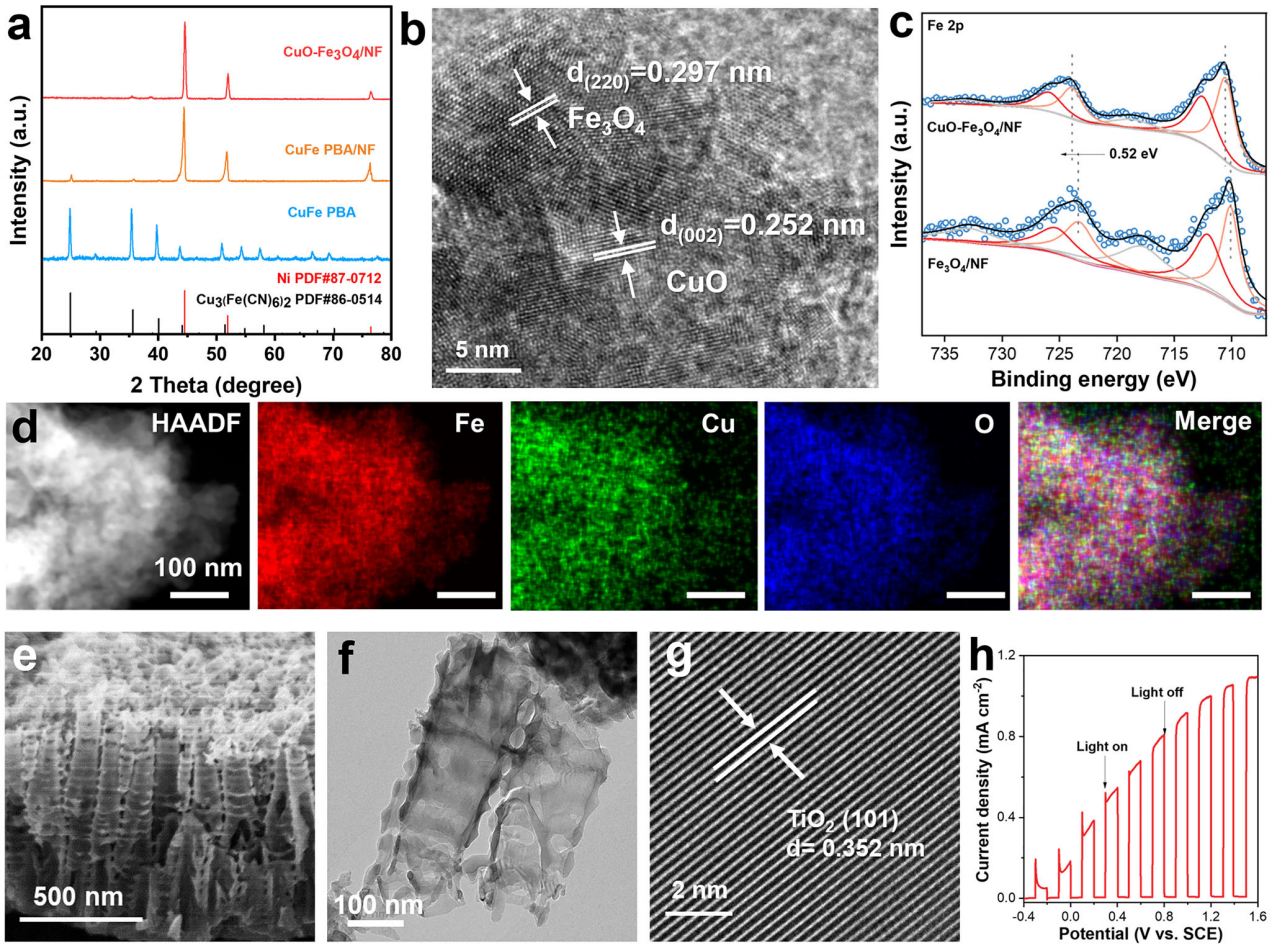
(a) XRD patterns of CuFe PBA, CuFe PBA/NF, and CuO‐Fe_3_O_4_/NF. (b) HRTEM image of CuO‐Fe_3_O_4_/NF. (c) Fe 2p XPS spectra of CuO‐Fe_3_O_4_/NF, and Fe_3_O_4_/NF. (d) HAADF‐STEM elemental mapping images of CuO‐Fe_3_O_4_/NF showing Fe (red), Cu (green), and O (blue). Scale bar: 100 nm. (e) SEM image, (f) TEM image and (g) HRTEM image of TiO_2_‐NTAs photoanodes. (h) LSV curve of TiO_2_‐NTAs measured in 0.02 M Na_2_SO_4_ and 0.02 M NaCl under chopped AM 1.5G illumination.

The cathodic CuO‐Fe_3_O_4_/NF electrode was synthesized by the calcination of CuFe prussian blue analogue (PBA)/Ni foam (CuFe‐PBA/NF), schematically illustrated in Figure S1. The morphologies of CuFe PBA/NF and CuO‐Fe_3_O_4_/NF were observed by scanning electron microscopy (SEM). As shown in Figure S2, the CuFe PBA particles appear as cube evenly distributed on the surface of NF. After calcination, the thermal cleavage of C‐N bonds in the CuFe PBA is accompanied by the incorporation of oxygen, resulting in the formation of a CuO‐Fe_3_O_4_ heterostructure on NF. The CuFe PBA, CuFe PBA/NF and CuO‐Fe_3_O_4_/NF were further verified by X‐ray diffraction (XRD) patterns (Figure 1a). All the characteristic diffraction peaks of CuFe PBA could be assigned to Cu_3_[Fe(CN)_6_]_2_ (JCPDS No. 86–0514). No other impurity diffraction peaks were detected in these samples, indicating the high purity of the products. The CuFe PBA/NF exhibited three high intensity peaks at 44.5°, 51.8°, and 76.3° can be attributed to (1 1 1), (2 0 0), (2 2 0) planes of nickel, respectively (JCPDS No. 87–0712). The other peaks can be corresponding to CuFe PBA, further indicating that CuFe PBA was successfully loaded on the nickel foam surface. For the CuO‐Fe_3_O_4_/NF, the peaks between 30.0° and 40.0° corresponds to (0 0 2), (1 1 1) planes of CuO (JCPDS No. 45–0937) and (3 1 1) plane of Fe_3_O_4_ (JCPDS No. 19–0629, Figure S3). Furthermore, high‐resolution transmission electron microscopy (HRTEM) image of CuO‐Fe_3_O_4_/NF reveals that the d‐spacing values of 0.252 nm and 0.297 nm correspond to the (0 0 2) plane of CuO and the (2 2 0) plane of Fe_3_O_4_, respectively (Figure 1b) [23, 24]. High‐angle annular dark‐field (HAADF) scanning TEM (STEM) and corresponding elemental mappings (Figure 1d) also showed that the elements Cu, Fe, and O are evenly distributed throughout the CuO‐Fe_3_O_4_ particle. Furthermore, the crystal planes labeled in the SAED pattern exhibit excellent consistency with those corresponding to the lattice fringes (Figure S4).

In addition, five elements (Cu, Fe, O, N, and C) were detected in the XPS survey spectrum of Cu‐Fe PBA/NF (Figure S5,). For CuO‐Fe_3_O_4_/NF, the enhanced signal of Cu, Fe, and O was observed in the survey spectrum, while no signal of N was detected. The changes observed in the high‐resolution XPS of Cu 2p confirmed that the CuO was truly produced by calcination of CuFe PBA (Figure S6a,c). The two peaks located at 933.8 and 953.7 eV are belonging to Cu 2p_3/2_ and Cu 2p_1/2_ of CuO, respectively (Figure S6c) [25]. In the high‐resolution spectrum of Cu 2p of CuFe PBA/NF, peaks located at 932.8 eV (2p_3/2_) can be assigned to Cu^0^ or Cu⁺, and peaks fitted at 935.5 eV belong to Cu^2^⁺ species. According to AES analysis of Cu LMM spectrum for CuFe PBA/NF, the peak for Cu⁺ with the kinetic energy of 915.8 eV is detected, while the peak for Cu0 at 918.3 eV is absent (Figure S7) [26]. The above results indicate that there are two valence states of copper in CuFe PBA, and all of them are converted to Cu^2^⁺ after calcination. The Fe 2p_3/2_ spectrum (Figure S6d) of CuO‐Fe_3_O_4_/NF can be split into two peaks with binding energies at 712.6 and 709.9 eV, which could be assigned to tetrahedral Fe(III), octahedral Fe(II), respectively [27]. Compared with the Fe 2p peak in Fe_3_O_4_/NF, the Fe 2p peak in CuO‐Fe_3_O_4_/NF shifted by 0.52 eV toward a higher binding energy (Figure 1c). Such corresponding shifts demonstrate that electron transfers from Fe to Cu occur in CuO‐Fe_3_O_4_ hetero‐phase interface, which is mainly attributed to the fact that Cu (electronegativity: 1.90) is more electronegative than Fe (electronegativity: 1.81) [28]. These results demonstrate the CuO and Fe_3_O_4_ formed the heterostructures in CuO‐Fe_3_O_4_/NF with distinct functionalities for the nitrite conversion.

Meanwhile, for TiO_2_‐NTAs photoanode, the XRD patterns corresponding to the standards of metallic Ti (JCPDS No. 44–1294) and TiO_2_ (JCPDS No. 21–1272) confirmed its composition (Figure S8). The obtained TiO_2_‐NTAs are neatly arranged, uniformly distributed, and have dimensions of 910 nm in length and 28 nm in thickness (Figure 1e,f). The HRTEM image reveals that the d‐spacing values of 0.352 nm correspond to the (1 0 1) plane of TiO_2_ (Figure 1g) [29]. The elemental mapping results demonstrated that Ti and O elements were uniformly distributed throughout the titanium dioxide nanotubes (Figure S9). The XPS analysis (Figure S10) was simultaneously performed to investigate the valence states of O and Ti elements, revealing two distinct oxygen species corresponding to Ti‐O (530.0 eV) and O‐H (532.0 eV), while the characteristic peaks at 458.7 eV (Ti 2p_3/2_) and 464.5 eV (Ti 2p_1/2_) were attributed to Ti^4^⁺ in TiO_2_ [30]. The valence and conduction band positions of TiO_2_‐NTAs were determined through UV–vis absorption spectroscopy and Mott‐Schottky analyses, revealing a bandgap energy of 2.9 eV with the valence band and conduction band positioned at +2.65 and −0.25 V (vs. NHE), respectively (Figure S11). The results of the LSV curves in 0.02 M Na_2_SO_4_ and 0.02 M NaCl under chopped light illumination (Figure 1h) prove the potential of TiO_2_‐NTAs as a photoanode for oxidizing Cl^−^ to Cl•.

**FIGURE 2:**
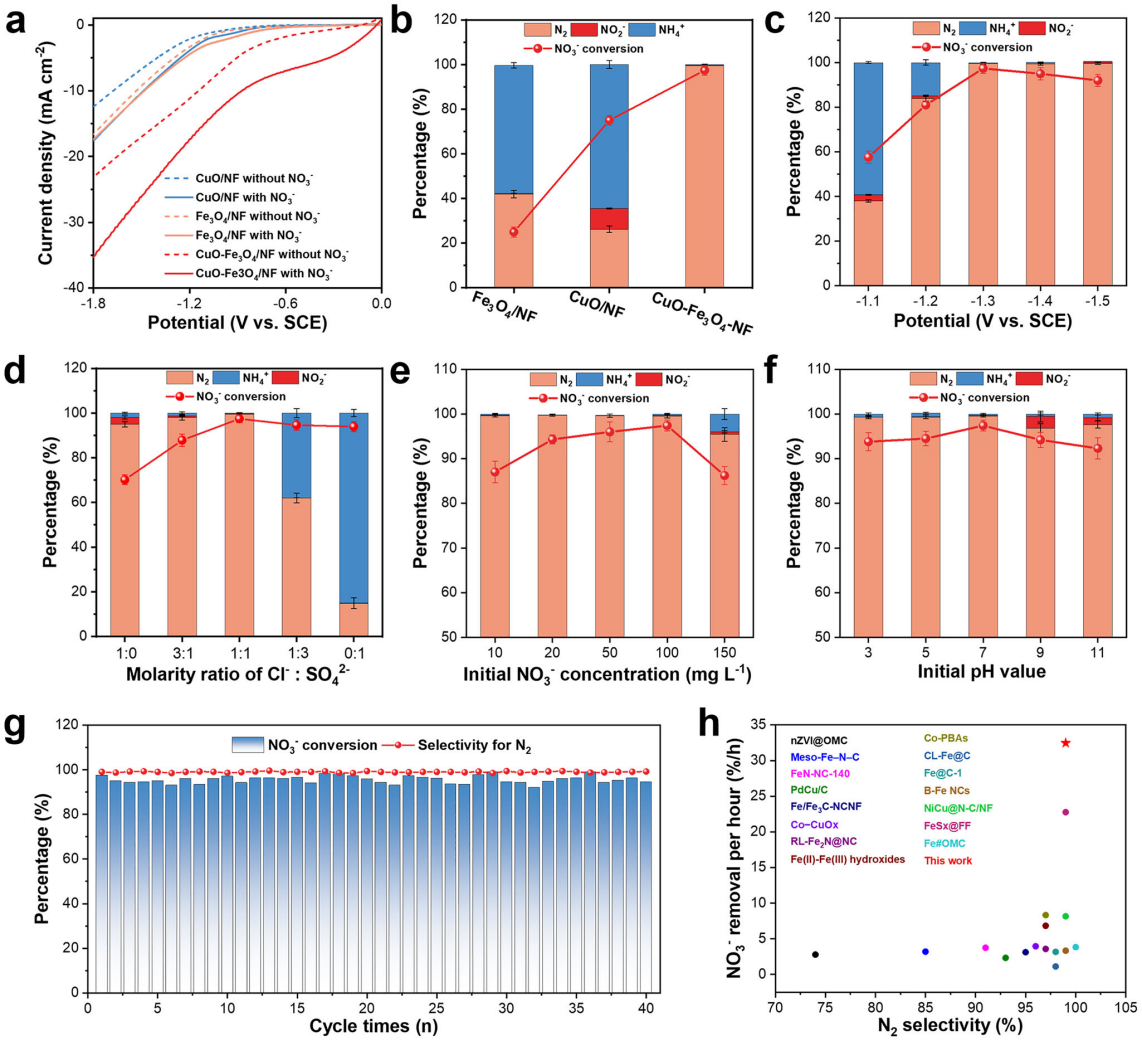
(a) LSV curves of CuO/NF, Fe_3_O_4_/NF, and CuO‐Fe_3_O_4_/NF in the presence (solid lines) and absence (dotted lines) of 100 mg/L of NO_3_^−^ in an electrolyte containing NaCl (0.02 M) and Na_2_SO_4_ (0.02 M). (b) Activity of different samples compared to that of CuO‐Fe_3_O_4_/NF under an applied potential of −1.3 V (vs. SCE), in an electrolyte containing NaCl (0.02 M), Na_2_SO_4_ (0.02 M), and 100 mg/L of NO_3_^−^ for 3 h. NO_3_RR activity over CuO‐Fe_3_O_4_/NF at different (c) voltages, (d) molar ratios of Cl^−^ to SO_4_^2^^−^, (e) initial concentrations of NO_3_^−^, and (f) initial pH values. Scale bars indicate standard deviations of the three independent tests. (g) NO_3_RR performance of CuO‐Fe_3_O_4_/NF over 40 cycles of repeated tests (each cycle continued for 3 h). (h) Comparison of the reaction time, selectivity of N_2_, and NO_3_^−^‐conversion rate of the synthesized catalyst for NO_3_RR with other catalysts reported in the literature.

The performance in nitrate removal to nitrogen of the as‐obtained catalysts was evaluated in a standard three‐electrode single‐cell configuration containing 0.02 M Na_2_SO_4_, 0.02 M NaCl, and additional 100 mg L^−1^ NO_3_^−^ using TiO_2_‐NTAs photoanode as counter electrode the under AM 1.5G illumination (100 mW cm^−2^) and SCE electrode as the reference electrode, respectively. To obtain a more precise comprehension of the function of CuO and Fe_3_O_4_ in nitrate reduction, the CuO/NF and Fe_3_O_4_/NF were prepared as the comparison working electrodes. First, linear scanning voltammetry (LSV) tests were performed in 0.02 M Na_2_SO_4_, 0.02 M NaCl electrolyte under neutral conditions (Figure 2a). The Fe_3_O_4_/NF had a higher current density than CuO/NF, indicating that H_2_O or H^+^ were more readily adsorbed by Fe_3_O_4_. When 100 mg L^−1^ NO_3_^−^ was added to the electrolyte, the cathodic current increased, and the current density of the CuO‐Fe_3_O_4_/NF after the nitrate addition was three and six times than that of CuO/NF and Fe_3_O_4_/NF, respectively, demonstrating that CuO‐Fe_3_O_4_/NF exhibited significantly higher catalytic activity toward nitrate electroreduction.

Nitrate reduction involves complex electron and proton transfer processes, we compared the electrochemical impedance spectroscopy (EIS) of three electrocatalysts (Figure S12). In the Nyquist diagram, the semicircle radius corresponds to charge transfer resistance, while the high‐frequency semicircle reflects electrode resistance. The results demonstrate that CuO‐Fe_3_O_4_/NF exhibits lower charge transfer resistance than both CuO/NF and Fe_3_O_4_/NF. In addition, we fitted the equivalent circuit diagram based on the results of electrochemical impedance spectroscopy (EIS). The interfacial charge transfer impedance value (Rct2) between CuO‐Fe_3_O_4_/NF and the electrolyte is the lowest, which indicates that it has the best adsorption and activation ability for nitrate molecules. Meanwhile, the smaller Rct1 value indicates that the formation of the heterojunction between CuO and Fe_3_O_4_ enhances the internal electron transfer ability. The electrocatalytic NO_3_RR performance was further evaluated by chronoamperometry, calibrated using Figure S13. After 3 h of electrolysis, Fe_3_O_4_/NF achieved a 25% nitrate conversion rate, which is significantly lower than the 75.0% conversion rate of CuO/NF, indicating superior NO_3_RR kinetics on Cu sites (Figure 2b). Although Fe_3_O_4_ acts as an effective H* donor, its ability to adsorb and activate nitrate is limited [31]. This is corroborated by the notably higher nitrite content in the electrolyte after CuO/NF electrolysis compared to Fe_3_O_4_/NF, consistent with CuO's established role in catalyzing nitrate‐to‐ammonia conversion. Both CuO/NF and Fe_3_O_4_/NF have very low nitrogen selectivity, and the electrolyte contain a large amount of NH_4_⁺. In the radical‐relay‐mediated PEC system, Cl^−^ is oxidized to Cl• by holes on the TiO_2_‐NTAs photoanode (Equation 1), and the generated Cl• further oxidizes NH_4_⁺ to nitrogen gas (Equation 2) [22]. The CuO/NF and Fe_3_O_4_/NF have relatively low current in the reaction, and it affects the rate of Cl• production at the TiO_2_‐NTAs photoanode resulting in a large amount of ammonium residue (Figure S14). Two‐phase composite material of CuO‐Fe_3_O_4_/NF has 97.4% nitrate conversion rate and more than 99.0% N_2_ selectivity, due to it combines the excellent adsorption and activation ability of CuO toward nitrate, and tandem H*‐mediated conversion of hydrogenation reaction to NH_4_^+^ at Fe_3_O_4_ sites via the spatial separation sites for the adsorption and transformation processes. To eliminate the interference of active site quantity variations on NO_3_RR performance, the electrochemically active specific surface areas (ECSA) of three comparative samples were evaluated. As shown in Figure S15, the electrochemical specific surface areas (ECSA) of the samples are as follows: CuO/NF (4.88 µF cm^−2^), Fe_3_O_4_/NF (5.33 µF cm^−2^), and CuO‐Fe_3_O_4_/NF (5.39 µF cm^−2^). The negligible difference in their electrochemically active specific surface areas indicates that the activity discrepancy originates from intrinsic activity variations rather than differences in active site abundance.

(1) $$h^{+}+Cl^{-}\to \mathrm{Cl}\cdot$$

(2) $$2N{H_{4}}^{+}+6\mathrm{Cl}\cdot \to N_{2}+6Cl^{-}+8H^{+}$$

The applied potential is an important parameter for affecting the reaction rates. As shown in Figure 2c, the conversion rate of nitrate increased gradually from 57.6% to 97.4% and maintained a good linear relationship when the cathode potential was decreased from −1.1 to −1.3 V (vs. SCE). The selectivity of nitrogen increased from 38.0% to 99.5% due to the increased reaction current density. The conversion of nitrate is slightly decreased to 92.0% and the selectivity of nitrogen is maintained above 99.0% when the cathode voltage further reduces to −1.5 V (vs. SCE). This phenomenon is attributed to the intensified hydrogen evolution side reaction and the obstruction of effective collisions between nitrate ions and active sites by the generated bubbles when the potential decreases from −1.3 to ‐1.5 V (vs. SCE) [32]. Then explore the effect of electrolyte type on the catalytic performance of nitrate reduction reaction (Figure 2d). By evaluating the nitrate removal rate and N_2_ selectivity in the electrolyte with different molar ratios of Cl^−^ and SO_4_^2^^−^ to determine the optimal electrolyte. The Cl^−^ is oxidized at the TiO_2_‐NTAs photoanode to form Cl•, which selectively oxidize ammonia to nitrogen. Therefore, adding appropriate amount of Cl^−^ can improve the selectivity of nitrate to nitrogen, which gave 15.0% selectivity for nitrogen when Cl^−^ was not added to the electrolyte. In SO_4_^2^^−^‐free electrolytes, the conversion rate of nitrate was only 70.0%, indicating that the addition of SO_4_^2^^−^ can promote the conversion of nitrate to ammonia [33]. The H* and Cl• relay system achieves the best nitrate conversion rate and nitrogen selectivity under the condition that the molecular ratio of Cl^−^ to SO_4_^2^^−^ is 1:1.

In order to investigate the differences of nitrate concentration in real wastewater, a series of nitrate concentration were prepared to analyze and evaluate the performance of the radical‐relay system (Figure 2e). As the initial NO_3_^−^ concentration increased from 10 to 100 mg L^−1^, nitrate conversion gradually increased from 87.0% to 97.4%, and nitrogen selectivity remained consistently above 99.0%. The conversion rate of nitrate was reduced to 86.2% when the initial concentration of nitrate was increased to 150 mg L^−1^. Therefore, the reduction of nitrate to N_2_ on the radical‐relay system was optimized to get high activity. The change of pH value in the reaction was monitored at different time points (Figure S16). The electrolyte pH value increased from 7.0 to 10.7 after reaction for half an hour, affecting the utilization efficiency of H*. The reduction of nitrate causes a change in pH, so the initial pH of the solution is an important condition that cannot be neglected in the actual nitrate reduction process. A series of electrolytes with different pH values (pH 3.0, 5.0, 7.0, 9.0, 11.0) were used to evaluate the electrocatalytic performance of the radical‐relay system (Figure 2f). It exhibited best nitrate conversion and nitrogen selectivity under neutral conditions. In the acidic system, it is easier to generate hydrogen bubbles on CuO‐Fe_3_O_4_/NF cathode which wrap around the surface of the active material and prevented sufficient contact between the active site and the electrolyte, thus slightly reducing the electrocatalytic performance. Competitive adsorption of hydroxide can also affect electron transfer from the electrode to nitrate under alkaline conditions. The nitrate conversion rate for the radial‐relay system is higher than 90.0% and the selectivity of N_2_ is higher than 95.0% at all pH values, indicating that the system maintains stable catalytic activity and selectivity in wastewater at different pH values for the possible actual application. The system's anti‐ion interference ability was also evaluated by introducing 100 ppm of three common anions into the electrolyte. The results are presented in Figure S17. These three ions had virtually no impact on the nitrate conversion rate and nitrogen selectivity, suggesting that the catalyst possesses a strong selective adsorption and activation capacity for nitrate.

To identify whether the detected Nitrogen species of NH_4_^+^ and N_2_ are from the NO_3_RR or other impurities in catalysts, the isotope tracing experiments are performed by using ^15^N‐labeled ^15^NO_3_^−^ as a reactant. After changing the reactant to ^15^NO_3_^−^ with 0.02 M Na_2_SO_4_, the triple ^1^H NMR peak for ^14^NH_4_⁺ alters to a double peak for ^15^NH_4_⁺ (Figure S18) [34]. The triple peak of ^14^NH_4_⁺ or the double peak of ^15^NH_4_⁺ could not be detected after adding Cl^−^, indicating that the detected NH_4_⁺ indeed originates from the NO_3_RR rather than impurities. In addition, cycling experiments were carried out to investigate the stability of the radical‐relay system during nitrate reduction. As can be seen from Figure 2g, after 40 consecutive electrolysis cycles (3 h per cycle) at −1.3 V (vs. SCE), the nitrate removal rate and the N_2_ selectivity did not change significantly. The nitrate conversion rate of 94.5% and nitrogen selectivity greater than 99.0% were still maintained in the 40th cycle experiment, indicating that the PEC system comprising CuO‐Fe_3_O_4_/NF cathode and TiO_2_‐NTAs photoanode has superb cycling stability. In addition, the time‐current curves of the first, 10th, 20th, 30th, and 40th cycles in the consecutive 40 cycles maintained almost the same trend, further demonstrating that the radical‐relay system has excellent long‐term cycling stability (Figure S19). The XRD pattern after 40 cycles of CuO‐Fe_3_O_4_/NF showed that the characteristic diffraction peaks of CuO and Fe_3_O_4_ were still observed, indicating that the crystal structure was well preserved (Figure S20a). The SEM image of CuO‐Fe_3_O_4_/NF demonstrated that the heterojunction particles exhibited no significant detachment or structural collapse (Figure S20b). No changes were observed in the valence state of the main active species by comparing the high‐resolution XPS spectra before and after the reaction (Figure S21). The TiO_2_‐NTAs photoanode also exhibits good stability. After forty cycles, neither the XRD patterns nor the XPS results show any changes compared with those before the reaction (Figure S22). Notably, to the best of our knowledge, the radical‐relay system shows an excellent catalytic efficiency for the NO_3_RR compared to previously reported state‐of‐the‐art electrocatalysts (Figure 2h and Table S1). Thus, due to its high stability and efficiency, the radical‐relay system shows high potential for NO_3_^−^ removal in wastewater treatment.

**FIGURE 3:**
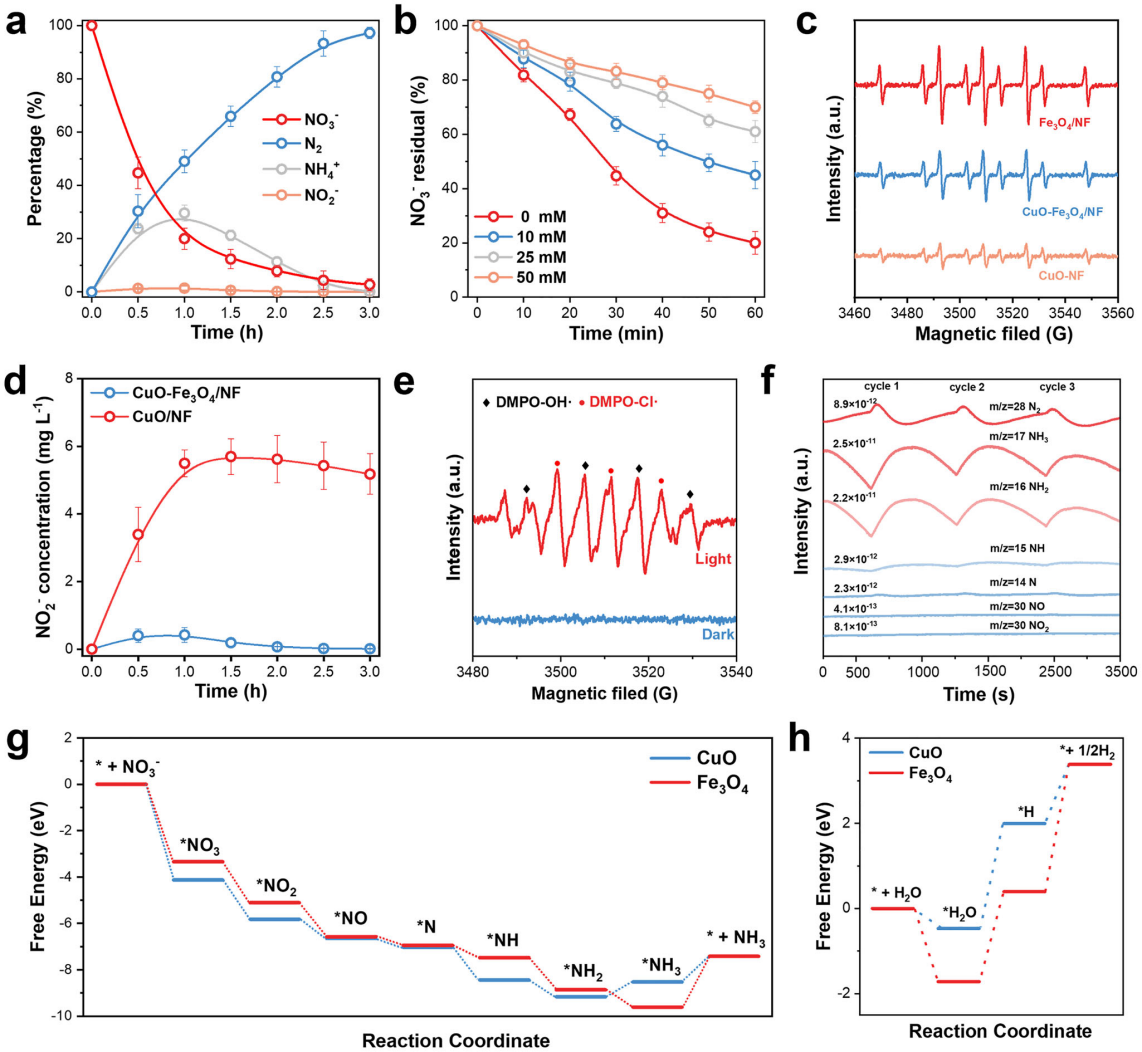
(a) Time‐dependent concentrations of NO_3_^−^, NO_2_^−^, NH_4_⁺, and N_2_ on the radical‐relay system. (b) Changes in residual nitrate percentage during NO_3_RR on CuO‐Fe_3_O_4_/NF under varying TBA concentrations. (c) DMPO spin‐trapping ESR spectra of CuO‐Fe_3_O_4_/NF, CuO/NF, and Fe_3_O_4_/NF at −1.3 V (vs. SCE). (d) Time‐dependent concentrations of NO_2_^−^ on CuO‐Fe_3_O_4_/NF and CuO/NF. (e) DMPO spin‐trapping ESR spectra of TiO_2_‐NTAs photoanode. (f) Online DEMS measurements of the NO_3_RR on the radical‐relay system. (g) Calculated Gibbs free energy changes for NO_3_RR leading to NH_3_ production at 0 V versus RHE. (h) Reaction energies for the adsorption of H_2_O and the production of H* and H_2_ on CuO (0 0 2) and Fe_3_O_4_ (2 2 0).

To explore the radial‐relay system for the removal of the NO_3_^−^ to N_2_, we monitor the changes in each nitrogen species over time during the reaction process (Figure 3a). The NO_3_^−^ concentration decreased from 100% to 20.0% within 1 h and further declined to 2.60% after 3 h. The concentration of NH_4_⁺ first increases and then gradually decreases. This is because the concentration of Cl• generated at the photoanode is insufficient to oxidize a large amount of NH_4_⁺ to N_2_ in time. Negligible NO_2_^−^ levels during the reaction confirmed its rapid conversion to NH_4_⁺ via H*. These quantification results demonstrate that the NO_3_RR is hypothesized to be NO_3_^−^ → NO_2_^−^ → NH_4_⁺ → N_2_ [35]. There are two deoxygenation paths for the reaction of nitrate adsorption on the electrode surface, one is the direct dissociation of oxygen by cathodic electrons, and the other is indirect dissociation by reactive H* generated on the active site [36]. Tert‐butanol (TBA), a specific hydrogen radical quencher, was used to determine whether the electrocatalytic nitrate reduction process was mediated by reactive H* or dominated by electronic regulation [37]. Different amounts of TBA were added to the electrolyte to suppress the concentration of active H* (from 0 to 50 mM). With the gradual increase of the addition of TBA, the conversion rate of nitrate decreased (Figure 3b). Compared with the case without addition of TBA, the conversion rate of nitrate was only 30.0% when the amount of TBA was 50 mM. The apparent rate constant of nitrate conversion over the first 1 h of CuO‐Fe_3_O_4_/NF was measured (Figure S23). It was found that the rate of nitrate reduction decreased significantly with the increasing of TBA concentration. This indicates that hydrogen adsorption plays a dominant role in the electrocatalytic reduction of nitrate in CuO‐Fe_3_O_4_/NF. Meanwhile, the formation of reactive H* radicals during the NO_3_RR was examined by electron spin resonance (ESR) experiments with a radical trapping reagent, 5,5‐dimethyl‐1‐pyrroline‐N‐oxide (DMPO). As shown in Figure S24, the nine peaks of the DMPO‐H* signal can be clearly distinguished without additional impurity peaks [15]. Furthermore, no signal peaks were observed in the blank control group, indicating the formation of active H* during the NO_3_RR. Additionally, a comparative assessment was performed to evaluate the ability of CuO/NF, Fe_3_O_4_/NF, and CuO‐Fe_3_O_4_/NF to generate active H*. As illustrated in Figure 3c, the Fe_3_O_4_/NF exhibits the most intense reactive H* signal, which substantiates that Fe_3_O_4_ is indeed an exceptional H* donor with superior performance. The adsorbed hydrogen provided by CuO is insufficient to rapidly convert NO_2_* into other intermediate products, while the competitive adsorption of NO_3_^−^ leads to a significant accumulation of NO_2_^−^ (Figure 3d). The DMPO was also used to trap the radicals generated on the side of the TiO_2_‐NTAs photoanode. As shown in Figure 3e, the signal peaks of Cl• and OH**·** were captured under the light‐on condition, while no radical signal peaks were detected under the dark condition [38]. This indicates that although two types of radicals (Cl• and OH**·**) are generated on the photoanode side, it is the chlorine radicals that play a role in selectively oxidizing ammonium to nitrogen. Furthermore, N‐tert‐butyl‐α‐phenylnitrone (PBN) was introduced into the electrolyte to scavenge chlorine radicals (Cl•) generated at the anode. As the concentration of PBN increased, the nitrogen selectivity decreased proportionally (Figure S25). These results conclusively validate the indispensable role of Cl• in the radical relay system.

Furthermore, the reaction intermediates and products were also captured by in situ electrochemical mass spectrometry (DEMS) to parse the entire reaction pathway over the catalyst. The gaseous intermediates and products formed at the cathode were immediately fed into an online mass spectrometer via a vacuum pump during the NO_3_RR test, and the corresponding mass‐charge ratio was then detected by mass spectrometry. The following major mass‐charge ratio (m/z) signals of 46, 30, 14, 15, 16, 17, 28 could be assigned to NO_2_*, NO*, N*, NH*, NH_2_*, NH_3_* and N_2_, respectively (Figure 3f). According to the DEMS findings, the nitrate reduction pathways (NO_3_* → NO_2_* → NO* → N* → NH* → NH_2_* → NH_3_ or NO_3_* → NO_2_* → NO* → N* → N_2_) have been identified. It can be judged that the predominant reaction path is NO_3_* → NH_3_ by comparing the relative intensities of N* and NH_3_; The weak NO_2_* signal peak confirmed the rapid conversion of NO_2_^−^ on the CuO‐Fe_3_O_4_/NF surface. As a result, the rapid transformation of intermediate products could be identified by the cascade conversion from NO_3_^−^ to NH_3_ and then to N_2_ in the radical‐relay system. In addition, density functional theory (DFT) calculations were employed to elucidate the catalytic mechanism of CuO and Fe_3_O_4_ in the NO_3_RR. We first calculated the free energy pathway of NO_3_RR from the result of EDMS to reveal the catalytic mechanism of the two facets, as shown in Figures S26 and S27. The pathway includes the adsorption of NO_3_^−^ to form NO_3_*, deoxygenation of the N species, hydrogenation of the N species, and desorption of the reduced species. The Gibbs adsorption energy (G_ads_) of NO_3_* on CuO (0 0 2) is −4.13 eV, lower than that of Fe_3_O_4_ (2 2 0) (−3.30 eV), suggesting that NO_3_^−^ tend to adsorb more on the CuO (0 0 2) facet (Figure 3g). The Gibbs free energy of CuO from NO_3_* to NO_2_* changes larger than Fe_3_O_4_, indicating the rate determination step (RDS) of NO_3_RR is faster on CuO. The adsorption energy of other intermediates on the CuO (0 0 2) surface is lower compared with that of Fe_3_O_4_ (2 2 0), indicating that the adsorption and activation takes place at CuO sites. In addition, the work functions of Fe_3_O_4_ and CuO were calculated using DFT calculation (Figure S28). The work function of CuO is 6.09 eV, which is greater than that of Fe_3_O_4_ (5.013 eV). This indicates that electrons flow from Fe_3_O_4_ to CuO, resulting in an increase in the electron cloud density at the Cu sites. This facilitates electron transfer from Cu sites into NO_3_^−^, promoting their activation. Meanwhile, the energy of HER on CuO (0 0 2) and Fe_3_O_4_ (2 2 0) was computed respectively (Figure 3h). The adsorption energy of Fe_3_O_4_ (−1.71 eV) for H_2_O significantly exceeds that of CuO (−0.47 eV). In addition, the energy barrier from H_2_O* to H* is smaller on Fe_3_O_4_ (2 2 0) and the H_2_ formation barrier on Fe_3_O_4_ was found to be 2.99 eV which also significantly higher than that of CuO (1.39 eV). This indicates that Fe_3_O_4_ is more likely to produce adsorbed hydrogen, which promotes the conversion from NO_2_^−^ to NH_4_⁺ via hydrogenation reaction. The differential charge densities of CuO (0 0 2) and Fe_3_O_4_ (2 2 0) after adsorbing NO_3_^−^ and H_2_O were also obtained, as shown in Figure S29. The adsorption of nitrate on CuO and Fe_3_O_4_ leads to an electron gain of 0.71 e^−^ and 0.58 e^−^, respectively, indicating that CuO has better pre‐activation ability for nitrate compared to Fe_3_O_4_, which is consistent with the results of the step‐by‐step Gibbs free energy diagram. When NO_3_^−^ is replaced by H_2_O, the situation is reversed: the electron transfer amount of the H_2_O molecule adsorbed on Fe_3_O_4_ (2 2 0) is 0.11 e^−^, higher than 0.042 e^−^ on CuO (0 0 2), indicating that water molecules have a stronger adsorption affinity for Fe_3_O_4_. These results demonstrated that the cathodic CuO‐Fe_3_O_4_ component achieves the strong adsorption ability at CuO sites and hydrogenation reaction at Fe_3_O_4_ sites for activating H_2_O to generate reductive atomic H*, then highly selectively generate the NH_4_^+^ by spatial decoupling adsorption‐transformation processes.

**FIGURE 4:**
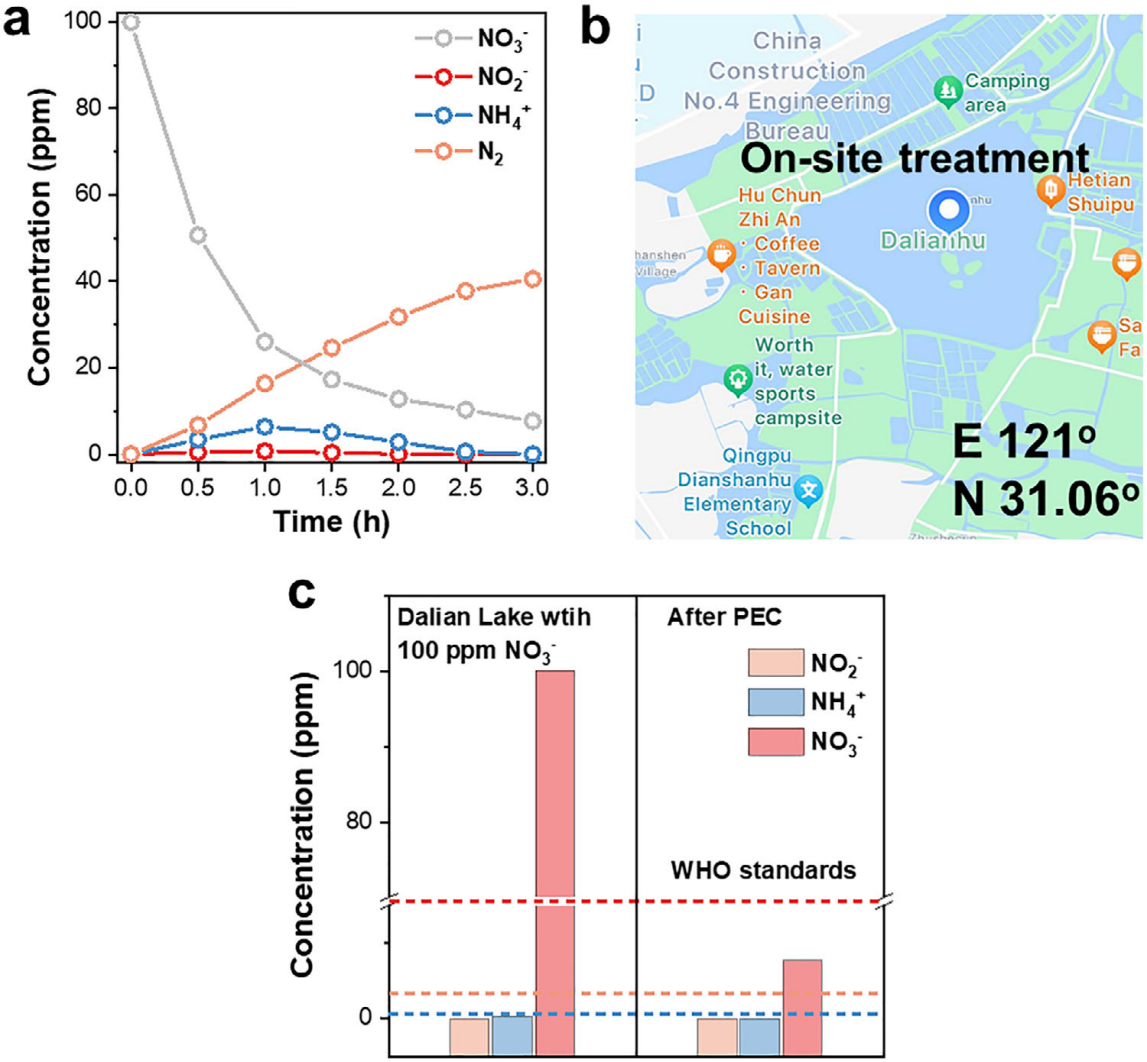
(a) The time‐dependent concentration changes of NO_3_^−^, NO_2_^−^, NH_4_⁺, and N_2_ during the treatment of natural water by the radical‐relay system. (b) Map of Dalian Lake (marked as located at 121° east longitude and 31.06° north latitude) (c) Comparison of the concentrations of different nitrogen‐containing species in natural water before and after treatment.

To further assess the denitrification efficacy of the free radical‐relay system under real aquatic conditions, natural water samples collected from Dalian Lake (Qingpu District, Shanghai) were subjected to treatment using this system, with the corresponding water quality parameters detailed in Table S2. Sodium nitrate, sodium sulfate and sodium chloride were added to the natural water body to increase the initial concentration of nitrate (to 100 mg L^−1^), electrical conductivity and chloride ion concentration. Although the accumulation of ammonia‐nitrogen could be detected in the early stage of actual natural water treatment, its concentration decreased as the reaction time increased (Figure 4a). The concentration of nitrite remained at an extremely low level, and the selectivity for nitrogen still reached 99.0%. The removal rate of nitrate still remains above 90%, which fully demonstrates the excellent performance of this system in the actual water treatment. After treatment (Figure 4b,c), the concentrations of residual nitrate, ammonium, and nitrite in the water were all lower than the standards set by the World Health Organization (WHO). It indicated that the radical‐relay system can remove the nitrate pollutants in the practical application.

## 3 Conclusion

In summary, we synthesized a CuO‐Fe_3_O_4_/Ni foam cathode and a TiO_2_‐NTAs photoanode, and then combined them to form a radical relay denitrification system. This system exhibits a high nitrate removal activity of 97.4% and nitrogen selectivity (>99.0%) at −1.3 V (vs. SCE). In addition, the system also exhibited ultra‐high stability, with no significant decrease in removal efficiency and selectivity after 40 cycles. The system also demonstrated excellent activity over a wide pH range, highlighting its potential for practical applications. Meanwhile, this system also achieves a high nitrate removal effect in real water bodies. In situ electrochemical investigations coupled with DFT calculations, unveiled the cooperative heterogeneous catalytic effects between CuO and Fe_3_O_4_ species as the key contributors to convert nitrate to ammonia. The preferential adsorption of NO_3_^−^ on the CuO (0 0 2) surface facilitated its conversion to NO_2_^−^, and Fe_3_O_4_ subsequently provided adsorbed hydrogen via water splitting for further hydrogenation reactions. The H* converts nitrite into a series of nitrogen‐containing intermediates and finally into ammonia. Ammonia is then oxidized by Cl• to generate nitrogen gas. This work presents a strategic approach to efficient denitrification, enabled by the unique relay catalysis mechanism between H* and Cl• radicals, addressing key challenges in conventional methods.

## Funding

This work was supported by National Key Research and Development Program of China (2020YFA0211004), National Natural Science Foundation of China (22176127, 22476131, 22376142, 22509135), Shanghai Eastern Talent Plan Leading Project (LJ2024115), the Shanghai Engineering Research Center of Green Energy Chemical Engineering (18DZ2254200), the China Postdoctoral Science Foundation (2024M762092) and Shanghai Pujiang Program by Science and Technology Commission of Shanghai Municipality (24PJA091).

## Conflicts of Interest

The authors declare no conflicts of interest.

## Supporting information




**Supporting File**: advs73507‐sup‐0001‐SuppMat.docx.

## Data Availability

The data that support the findings of this study are available from the corresponding author upon reasonable request.
