# Supplementary material for: Synergetic Relay Between Atomic Hydrogen and Chlorine Radicals Enables Efficient Nitrate‐to‐Nitrogen Gas Conversion
Source: Adv Sci (Weinh). 2025 Dec 23;13(13):e22147. doi: 10.1002/advs.202522147 (PMC12955995; doi:10.1002/advs.202522147)
Supplement: Supplementary file 1 — Supporting File: advs73507‐sup‐0001‐SuppMat.docx. [file ADVS-13-e22147-s001.docx]

Supporting Information

Synergetic Relay between Atomic Hydrogen and Chlorine Radicals Enables Efficient Nitrate-to-Nitrogen Gas Conversion

*Yongjie Wang, Ying Tao, Chi Zhang, Shan Hu, Yuxin Shi, Jixing Wang, Jiacheng Zhang, Chen Cheng, Ying Wang, Guisheng Li,* Zichao Lian,* Dieqing Zhang**

W. Wang, Dr. Y. Tao, Dr. C. Zhang, Dr. S. Hu, Y. Shi, J. Wang, Prof. D. Zhang
The Education Ministry Key Lab of Resource Chemistry, Joint International Research Laboratory of Resource Chemistry of Ministry of Education, Shanghai Key Laboratory of Rare Earth Functional Materials, and Shanghai Frontiers Science Center of Biomimetic Catalysis
Shanghai Normal University
Shanghai 200234, China
E-mail: dqzhang@shnu.edu.cn.

Dr. J. Zhang, Prof. G. Li, Prof. Z. Lian
School of Materials and Chemistry
University of Shanghai for Science and Technology
Shanghai 200234, China
Email: liguisheng@shnu.edu.cn; [zichaolian@usst.edu.cn](mailto:zichaolian@usst.edu.cn)

Dr. C. Chen, Prof. Y. Wang
State Key Laboratory of Water Pollution Control and Green Resource Recycling, Shanghai Institute of Pollution Control and Ecological Security, College of Environmental Science and Engineering
Tongji University
Shanghai, 200092, China

**Table of Contents**

1. **Experimental Procedures**
2. **Results and Discussion (Figures S1-S29 and Tables S1-S2)**
3. **References**

**Text S1.** Chemicals.

FeCl_3_·6H_2_O (≥99%) and CuCl_2_·2H_2_O (≥99%) were purchased from Aladdin Ltd. (Shanghai, China). Polyvinylpyrrolidone (MW~48000) and Sodium citrate dihydrate (C_6_H_5_Na_3_O_7_·2H_2_O) were purchased from Beijing Mairuida Technology Co., LTD. Potassium ferricyanide (K_3_Fe(CN)_3_) was purchased from Shanghai Runjie Chemical Reagent Co., LTD (China). Titanium sheets (0.3 mm in thickness, 99.5% in purity) were purchased from Shanghai Right Titanium Industry Co., Ltd. Analytical grade ammonium fluoride, phosphoric acid, acetone, and ethanol were obtained from the Aladdin Company and used without further purification. All reagents were used directly without further treatment. Deionized water (DIW) with 18.2 MΩ was used in all experimental preparations and measurements.

**Text S2.** Synthesis of CuO-Fe_3_O_4_/NF.

0.1 mmol of PVP, 2.1 mmol of sodium citrate dihydrate, and 1.5 mmol of copper (II) chloride dihydrate were dissolved in 300 mL of deionized water, and the resulting solution was labeled A. 0.5 mmol potassium ferricyanide dissolved in 200 mL of deionized water, the solution obtained marked as B. The stirring continued for 15 minutes after adding solution A drops to solution B. Nickel foam (NF, 1 × 2 cm^2^) was inserted into the solution to age for 24 h. Before utilization, NF was cleaned by successive ultrasonic washings in 5 M HCl, deionized (DI) water, and ethanol. Afterward, modified NF named CuFe PBA/NF was isolated after age and washed repeatedly with ethanol, DI water, and dried for 24 h at 60 °C. The Fe PBA/NF was synthesized in the same way as above, simply replacing copper chloride with ferric chloride. The CuFe PBA was calcined at 350 ^o^C for two hours under argon atmosphere and the heating rate was 5 ^o^C/min to obtain CuO-Fe_3_O_4_/NF. Fe_3_O_4_/NF was obtained by calcination of Fe PBA/NF under the same conditions.

**Text S3.** Synthesis of TiO_2_ Nanotube Arrays (TiO_2_-NTAs).

TiO_2_-NTAs electrode was fabricated with the electrochemical anodic oxidation method reported in previous work. In a typical process, a 0.3 mm thick polished titanium sheet was cut into several of rectangle pieces with size of 1×2 cm^2^. The titanium pieces were then cleaned by ultrasonication in acetone, ethanol and deionized water sequentially for 15 min each. The cleaned titanium piece and platinum foil were used as the anode and cathode, respectively. An aqueous solution containing 0.2 mol L^−1^ NH_4_F and 0.1 mol L^−1^ H_3_PO_4_ was used as the electrolyte for the anodic oxidation process. Under a constant voltage of 20 V supplied by a DC constant voltage power for 7 h, amorphous TiO_2_-NTAs was obtained. The as-prepared TiO_2_-NTAs was rinsed with deionized water and dried in air before the use.

**Text S4.** Material Characterizations.

X-ray diffraction (XRD) measurements were carried out in a parallel mode (2θ varied from 20° to 80°) using a Rigaku Dmax-3C Advance X-ray diffractometer (Cu Kα radiation, λ=1.5406 Å). Transmission electronic micrograph (TEM), high resolution transmission electron microscopy (HRTEM) and selected area electronic diffraction (SAED) were recorded on a JEOL JEM-2100 working at 200 kV. X-ray photoelectron spectroscopy (XPS) was used to analyze the electronic states by a Thermo Fisher Scientific ESCALAB 250Xi. UV-vis absorption spectra were recorded on LengGuang 759S spectrophotometer (Shanghai). Electron spin resonance spectra (MICROESR STANDARD V 3.0, Bruker) were used to record the active free radical signal. Isotope labeling ^1^H NMR spectra was performed using a 600 MHz NMR spectrometer (Bruker Advance 3 HD 600 MHz).

**Text S5.** Electrochemical Measurements.

The electrocatalytic reduction of nitrate occurred in a 50 mL single chamber electrochemical reaction cell with constant voltage mode. All the electrochemical tests were performed on a CHI 660D electrochemical analyzer system. The work electrode, reference electrode (Saturated calomel electrode), and counter electrode (TiO_2_-NTAs) were employed, respectively. The electrolyte comprised 35 mL of a mixed solution of 0.02 M Na_2_SO_4_ and 0.02 M NaCl with 100 ppm NO_3_⁻. The potential range for measuring NO_3_⁻ removal and selectivity of nitrogen ranged from -1.1 V to -1.5 V (vs SCE), with intervals -0.1 V. The LSV tests were conducted at 0 to -1.7 V (vs SCE) at 10 mV/s scan rate. The EIS analysis was recorded at 10^-1^ Hz to 10^6^ Hz with 5 mV amplitude. In Na^15^NO_3_ isotope labeling experiments, a 35 mL solution containing 0.02 M Na_2_SO_4_ + 0.02 M NaCl with 100 ppm ^15^NO_3_⁻ served as the NO_3_RR electrolyte. Subsequently, 0.3 mL of the electrolyte and 0.3 mL of deuterium oxide (D_2_O) were added to the nuclear magnetic resonance (NMR) tube for further detection using NMR at 600 MHz.

**Text.S6.** *In-situ* differential electrochemical mass spectrometry (DEMS) analysis.

For the online DEMS tests, 100 mg/L NO_3_⁻ and 0.02 mol/L Na_2_SO_4_ and 0.02 mol/L NaCl as electrolyte was flowed using a specially-made peristaltic pump-driven electrochemical cell at a constant speed. Linear sweep voltammetry (LSV) tests were conducted from 0 to -1.7 V (vs SCE) at 10 mV/s scan rate after the baseline kept steady. Accordingly, the differential mass signals appeared when the gaseous products formed on the electrode surface. The mass signal returned to baseline when the electrochemical LSV process was over. To avoid accidental errors, three LSV tests were subsequently conducted under the same conditions.

**Text S7.** Detection of nitrogen species.

**Determination of nitrate-N.** Nitrate concentration was determined by ion chromatography (Dionex, thermos scientific).

**Determination of ammonia-N.** Ammonia-N was determined using Nessler’s reagent as the color reagent. First, a 0.5 mL electrolyte was taken out from the electrolytic cell and diluted to 5 mL. Next, 0.1 mL potassium sodium tartrate solution (ρ=500 g/L) was added and mixed thoroughly, then 0.15 mL Nessler’s reagent was put into the solution. The absorption intensity at a wavelength of 420 nm was recorded after waiting for 10 minutes. The concentration-absorbance curve was calibrated using a series of standard ammonium chloride solutions and the ammonium chloride crystal was dried at 105-110 ^o^C for 2 h in advance.

**Determination of nitrite-N.** A mixture of p-aminobenzenesulfonamide (20 g), N-(1-Naphthyl) ethylenediamine dihydrochloride (1 g), ultrapure water (250 mL) and phosphoric acid (50 mL, ρ = 1.70 g/mL) was used as a color reagent. A 1 mL of electrolyte was taken out from the electrolytic cell. Next, 1 mL color reagent was added into the aforementioned solution and mixed uniformity and diluted to 50 mL to detection range, and the absorption intensity at a wavelength of 540 nm was recorded after waiting for a period of time. The concentration-absorbance curve was calibrated using a series of standard sodium nitrite solutions.

**Text S8.** Methodology for performance evaluation.

The conversion percentage of nitrate (CP(NO_3_^−^)) and the product selectivity of nitrite(S(NO_2_^–^) and ammonium (S(NH_4_^+^)) and nitrogen gas (S(N_2_)) were calculated according to the following formulas:

The nitrate conversion (C (NO_3_ ^−^ )) is determined by the Equation 1:

$\text{CP}\left( \text{NO}_{\text{3}}^{\text{-}} \right)\text{\%=}\frac{\text{C}_{\text{0}}\left( \text{NO}_{\text{3}}^{\text{-}}\text{-N} \right)\text{-}\text{C}_{\text{t}}\text{(}\text{NO}_{\text{3}}^{\text{-}}\text{-N)}}{\text{C}_{\text{0}}\text{(}\text{NO}_{\text{3}}^{\text{-}}\text{-N)}}\text{×100\%}$ (1)

The selectivity of products including nitrite, ammonia, and nitrogen are obtained as follows:

$\text{S}\left( \text{NO}_{\text{2}}^{\text{-}} \right)\text{\%=}\frac{\text{C}_{\text{t}}\left( \text{NO}_{\text{2}}^{\text{-}}\text{-N} \right)\text{-}\text{C}_{\text{0}}\text{(}\text{NO}_{\text{2}}^{\text{-}}\text{-N)}}{\text{∆C(}\text{NO}_{\text{3}}^{\text{-}}\text{-N)}}\text{×100\%}$ (2)

$\text{S}\left( \text{NH}_{\text{4}}^{\text{+}} \right)\text{\%=}\frac{\text{C}_{\text{t}}\text{(}\text{NH}_{\text{4}}^{\text{+}}\text{-N)}}{\text{∆C(}\text{NO}_{\text{3}}^{\text{-}}\text{-N)}}\text{×100\%}$ (3)

$\text{S}\left( \text{N}_{\text{2}} \right)\text{\%=}\frac{\text{∆C}\left( \text{NO}_{\text{3}}^{\text{-}}\text{-N} \right)\text{-}\text{C}_{\text{t}}\left( \text{NO}_{\text{2}}^{\text{-}}\text{-N} \right)\text{-}\text{C}_{\text{t}}\text{(}\text{NH}_{\text{4}}^{\text{+}}\text{-N)}}{\text{∆C(}\text{NO}_{\text{3}}^{\text{-}}\text{-N)}}\text{×100\%}$ (4)

where C_0_(NO_3_^−^-N) is represented for the initial NO_3_^−^-N concentration and C_t_ (NO_3_^−^-N) is the concentration of nitrate at given reaction time and ΔC (NO_3_^−^-N) is represented for the change of NO_3_^−^-N at a given time. C_0_(NO_2_^−^-N) is the initial concentration of nitrite in the electrolyte. C_t_ (NO_2_^–^-N) and C_t_ (NH_4_^+^-N) are represented for the concentration of nitrite and ammonium at the given time. Due to the ultralow amount, other gaseous products (e.g., N_2_O, NO, NO_2_, NH_2_OH, and H_2_) are ignored for calculation.

**Text S9.** DFT calculations.

All spin-polarized DFT calculations were implemented in the Vienna *ab initio* Simulation Package (VASP) package.^[1]^ The exchange-correlation interactions were modeled by the functional of Perdew, Burke, and Ernzerhof (PBE) within the generalized gradient approximation (GGA), and the projector augmented-wave method was carried out to describe the ion-electron interactions.^[2]^ The Brillouin zone was sampled by 3×3×1 k-points by means of Monkhorst-Pack scheme with the cutoff of 420 eV during the structural relaxation. The convergence criteria for energy and force were set as 10^-5^ eV and 0.01 eV A^-1^, respectively. Along the perpendicular direction, a vacuum space at least 25 Å was added to eliminate the effects of periodic structure, and the empirical density functional dispersion (DFT-D3) correction was utilized to include van der Waals (vdWs) interaction between adsorbate and interface.^[3]^ Based on Nørskov et al’s theory, the change of Gibbs free energy for each elementary reaction was calculated based on computational hydrogen electrode (CHE) model, referred as $\Delta G= \Delta E+ \Delta ZPE-T\Delta S$, where Δ*E* is the adsorption electronic energy, while Δ*ZPE* and TΔ*S* represent zero-point energy correction and entropy contribution at 298.15 K. For the gas molecules, the entropy was taken from NIST database. For the adsorption intermediates, only vibrational entropy was considered, which was calculated from the DFT calculated vibrational frequencies.^[4]^

**Figures and Tables**

**
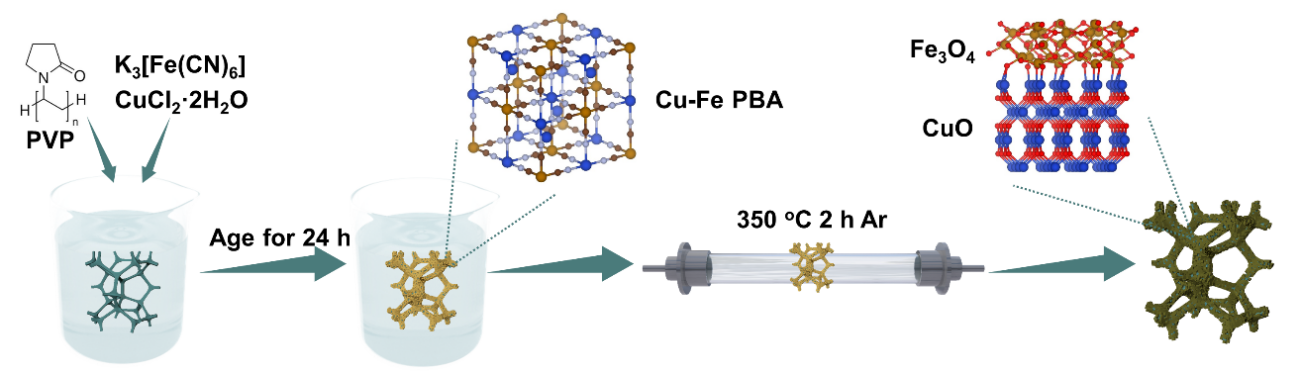
**

**Figure S1.** Schematic illustration of synthetic procedures for CuO-Fe_3_O_4_/NF electrode.

**
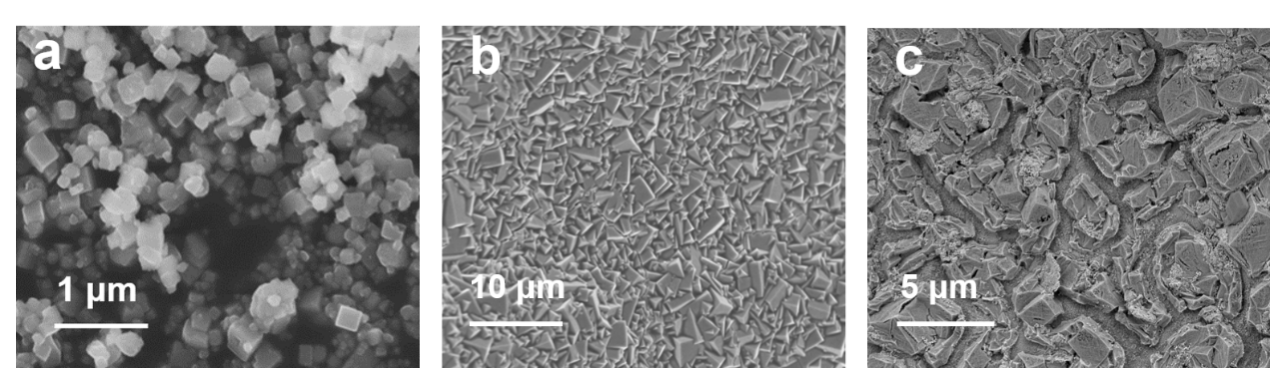
**

**Figure S2.**SEM images for CuFe PBA (a) CuFe PBA/NF (b) and CuO-Fe_3_O_4_/NF (c).


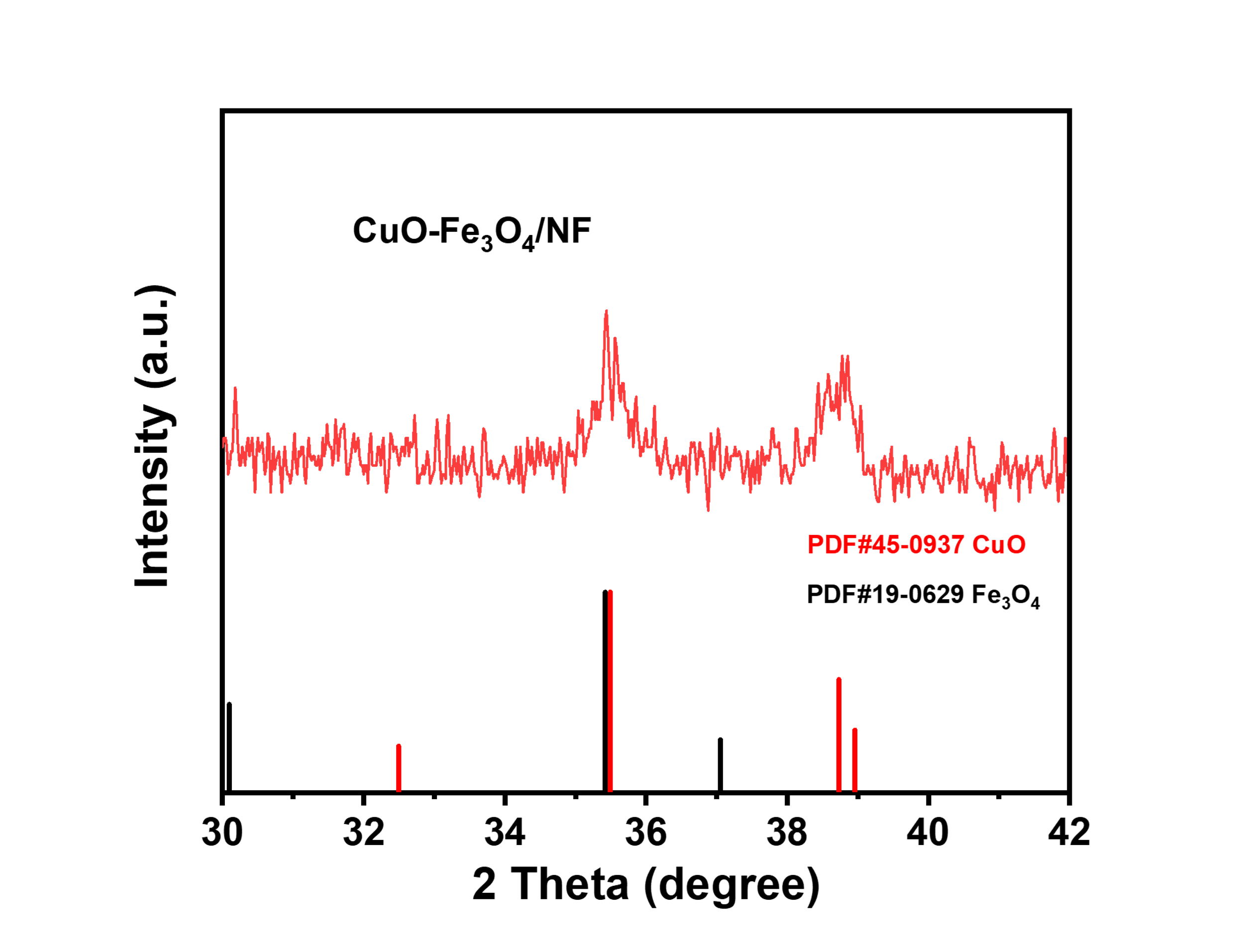


**Figure S3.** XRD pattern partial enlarged view of CuO-Fe_3_O_4_/NF

*
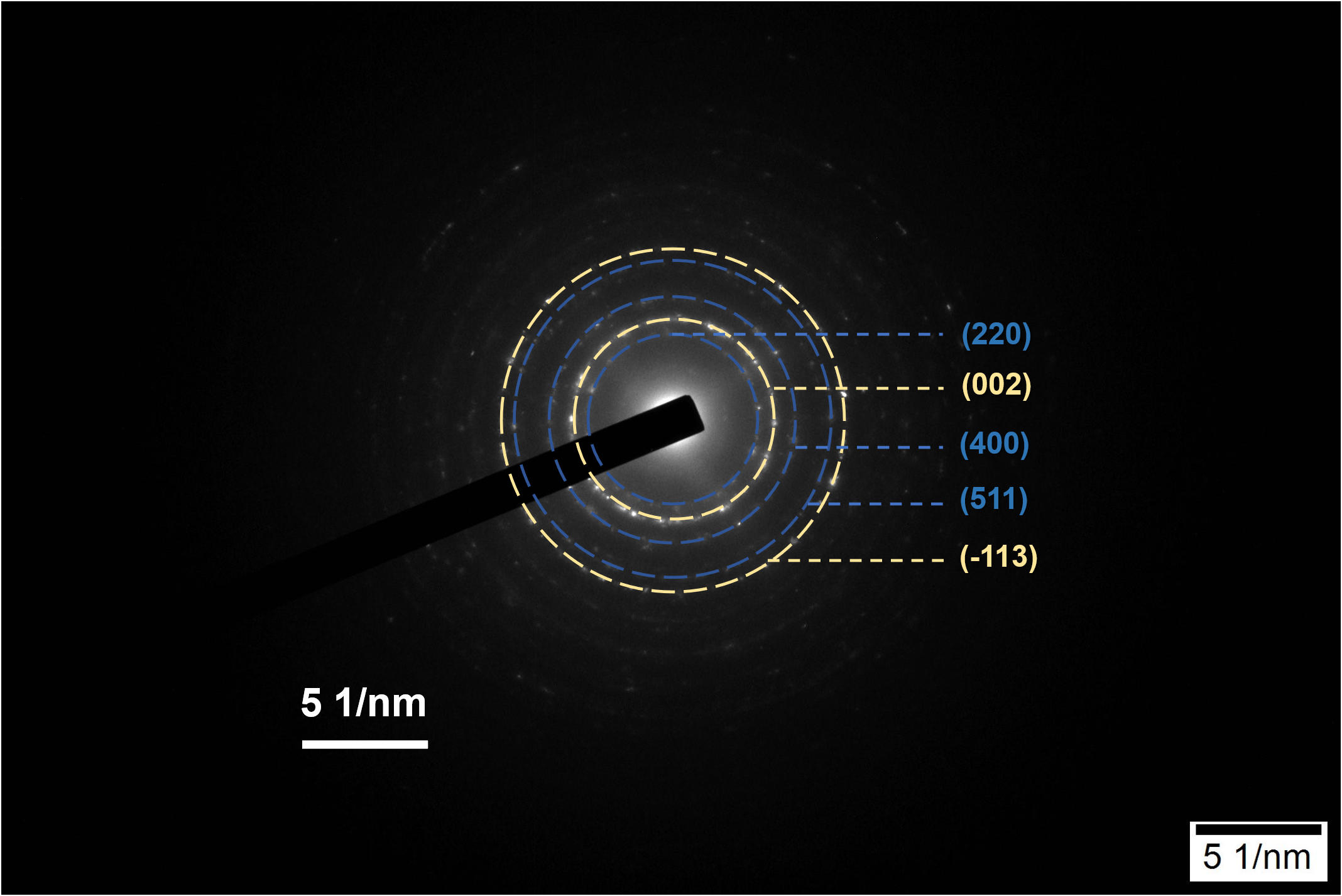
*

**Figure S4.** SAED pattern of CuO-Fe_3_O_4_/NF. The blue dotted line corresponds to the diffraction ring of Fe_3_O_4_, and the yellow dotted line corresponds to the diffraction ring of CuO.


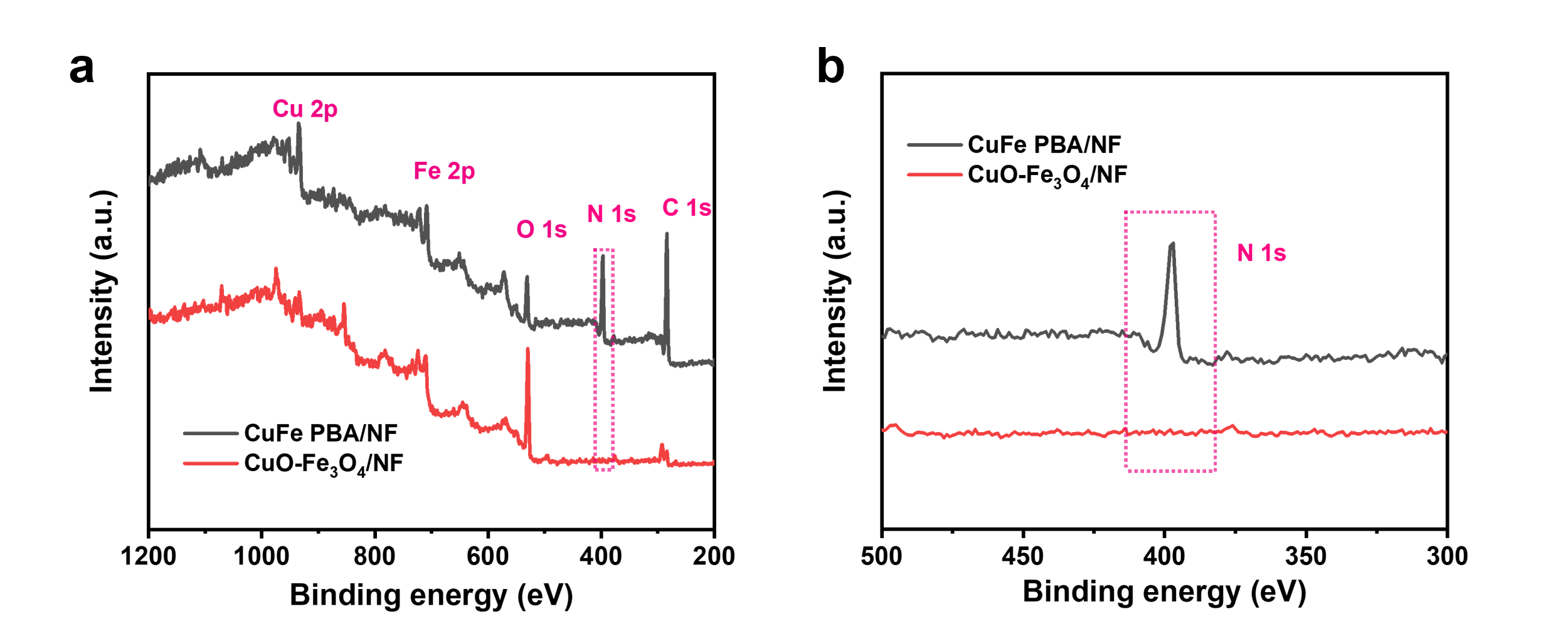


**Figure S5.** XPS spectra of CuFe PBA/NF and CuO-Fe_3_O_4_/NF a) and partial enlarged view b).


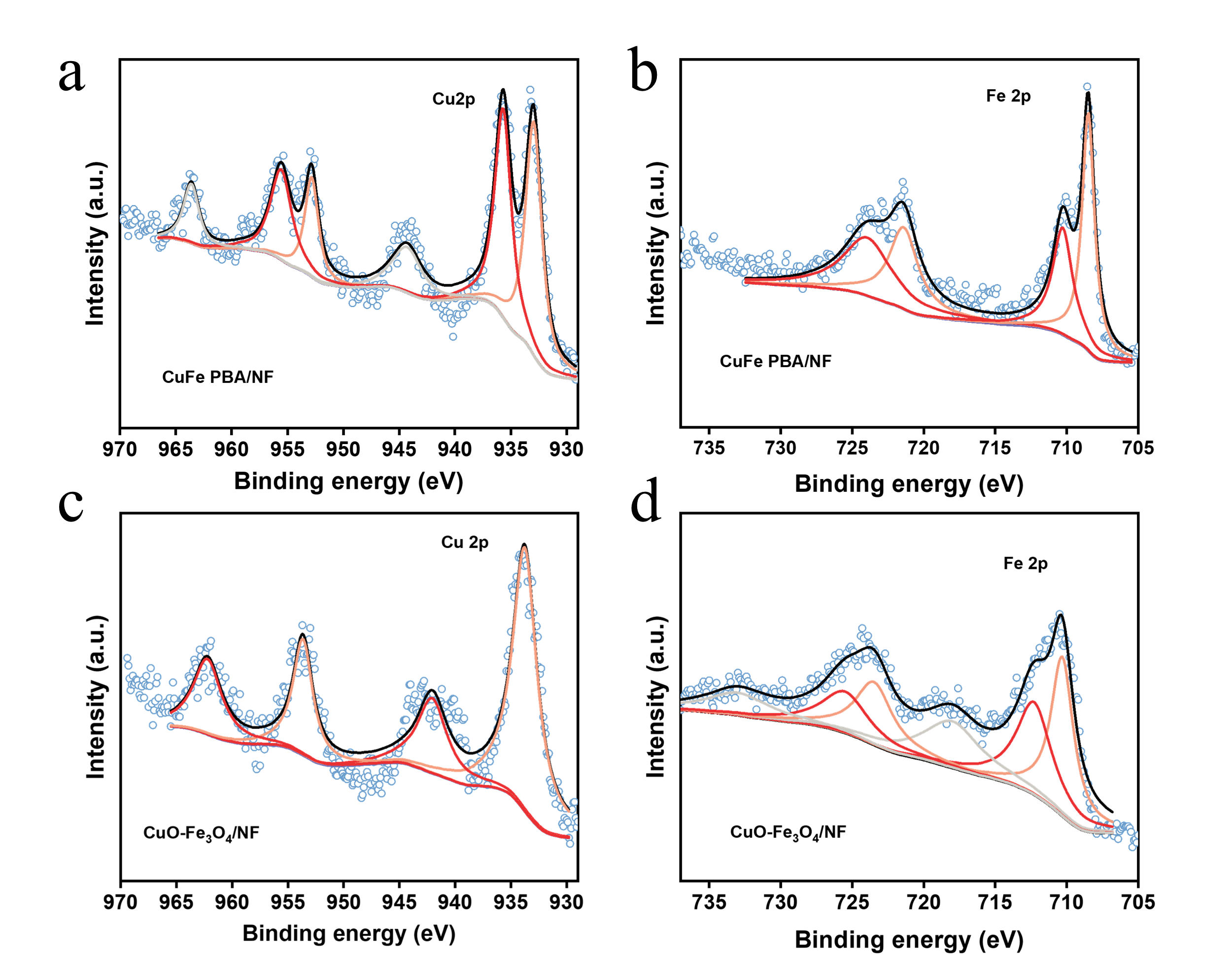


**Figure S6.** (a) high-resolution Cu 2p XPS spectra of CuFe PBA/NF, (b) high-resolution Fe 2p XPS spectra of CuFe PBA/NF, (c) high-resolution Cu 2p XPS spectra of CuO-Fe_3_O_4_/NF, and (d) high-resolution Fe 2p XPS spectra of CuO-Fe_3_O_4_/NF .


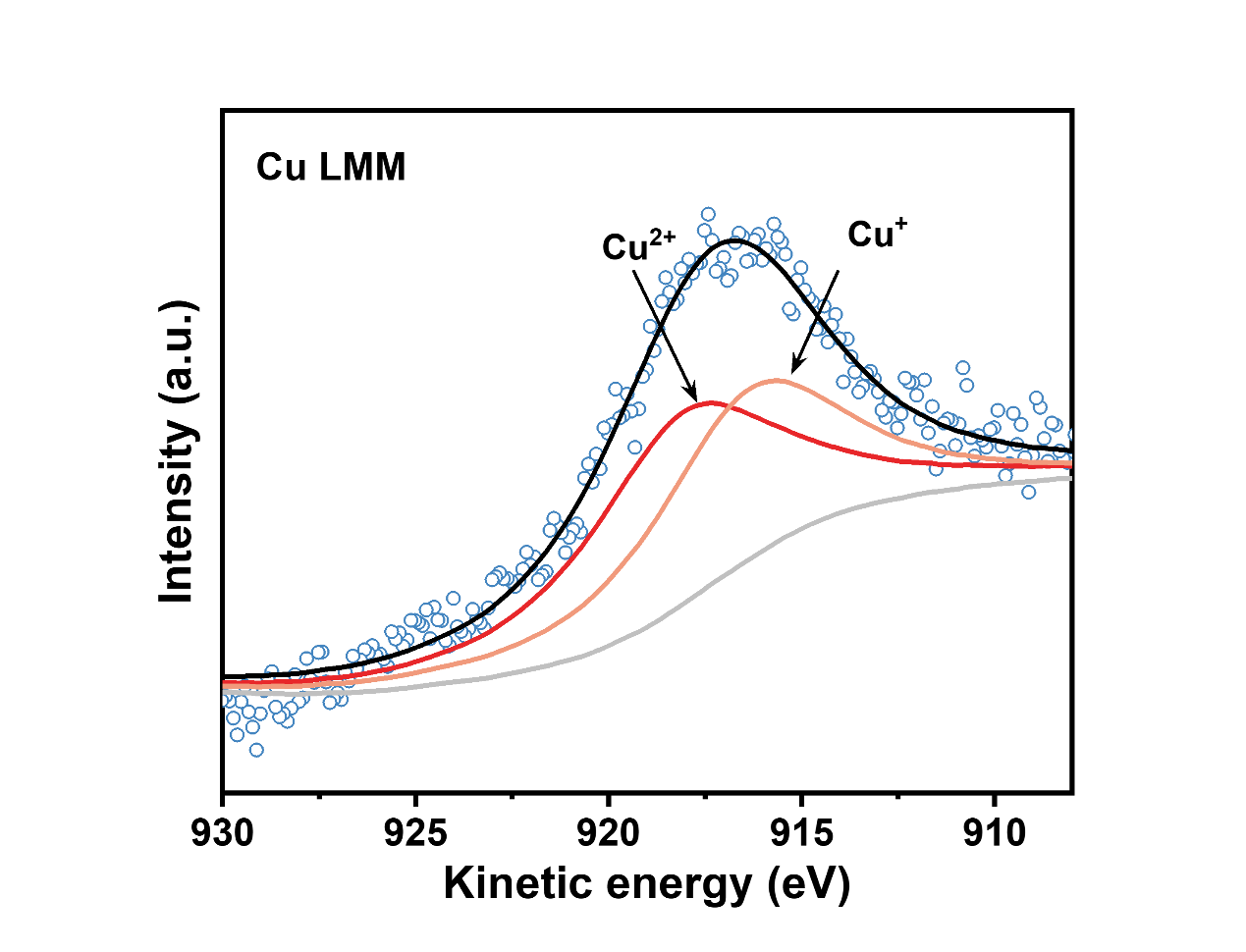


**Figure S7.** Cu LMM AES spectra of CuFe PBA/NF.

**Figure S8.** The XRD pattern of TiO_2_-NTAs.


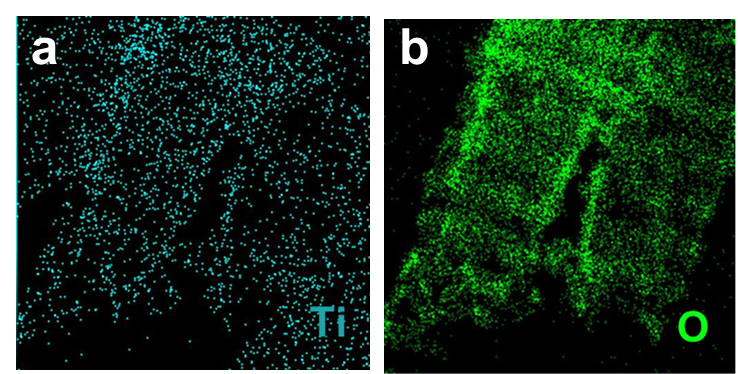


**Figure S9.** The elemental mapping images of TiO_2_-NTAs photoanode.


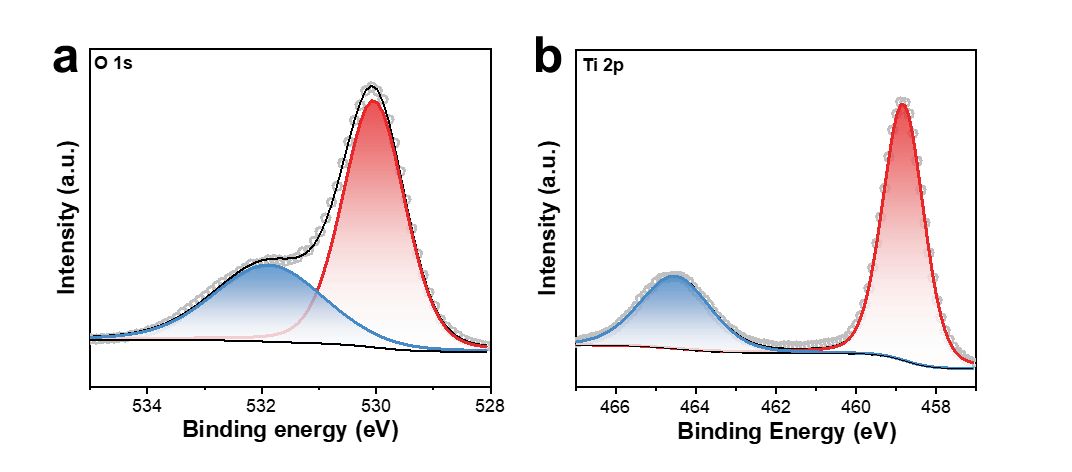


**Figure S10.** (a) O 1s, (b) Ti 2p XPS spectra of TiO_2_-NTAs.


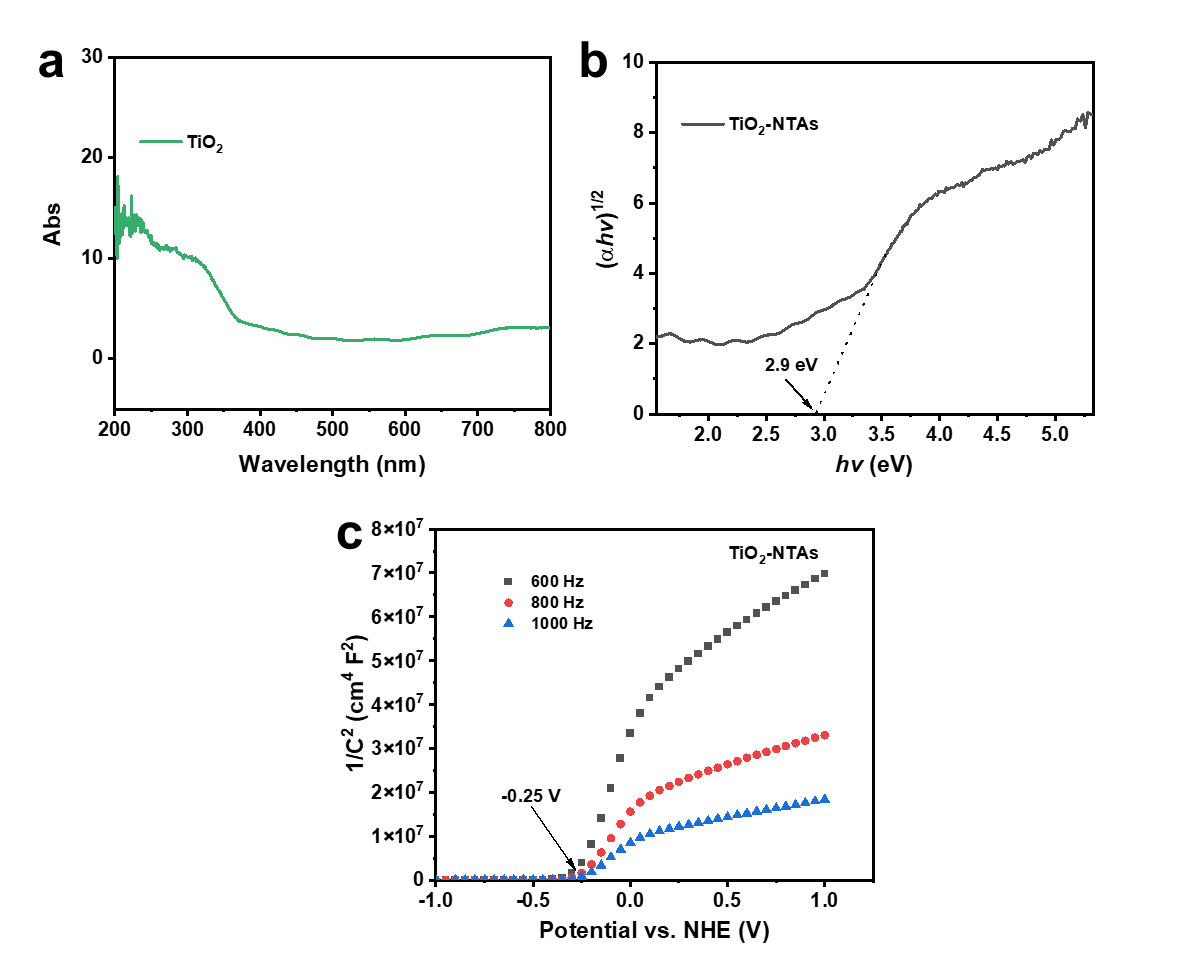


**Figure S11.** (a) UV-Vis spectrum, (b) Tauc plot, and (c) Mott-Schottky curve of TiO_2_-NTAs.


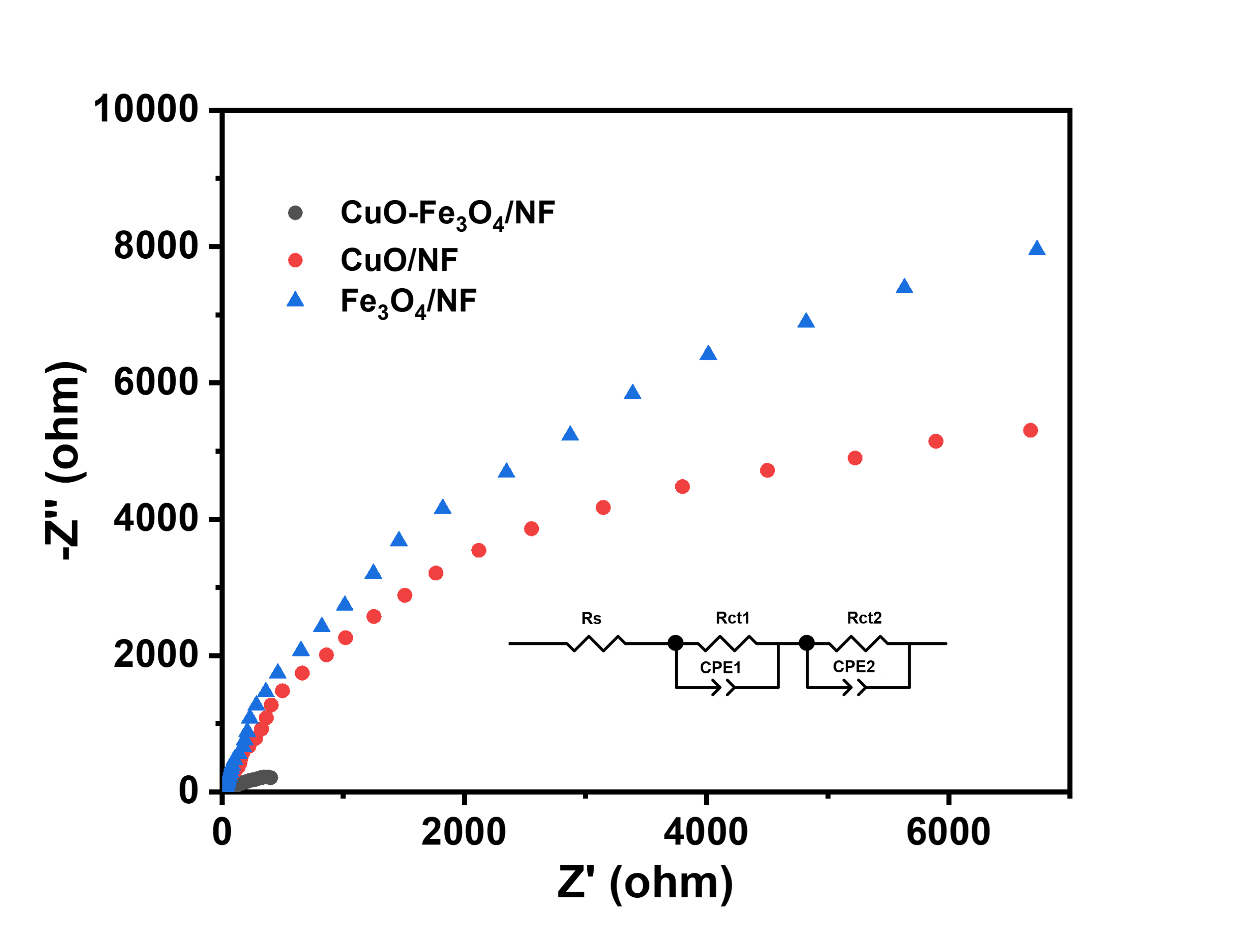


**Figure S12.** EIS spectra of CuO-Fe_3_O_4_/NF , CuO/NF , and Fe_3_O_4_/NF cathodes.


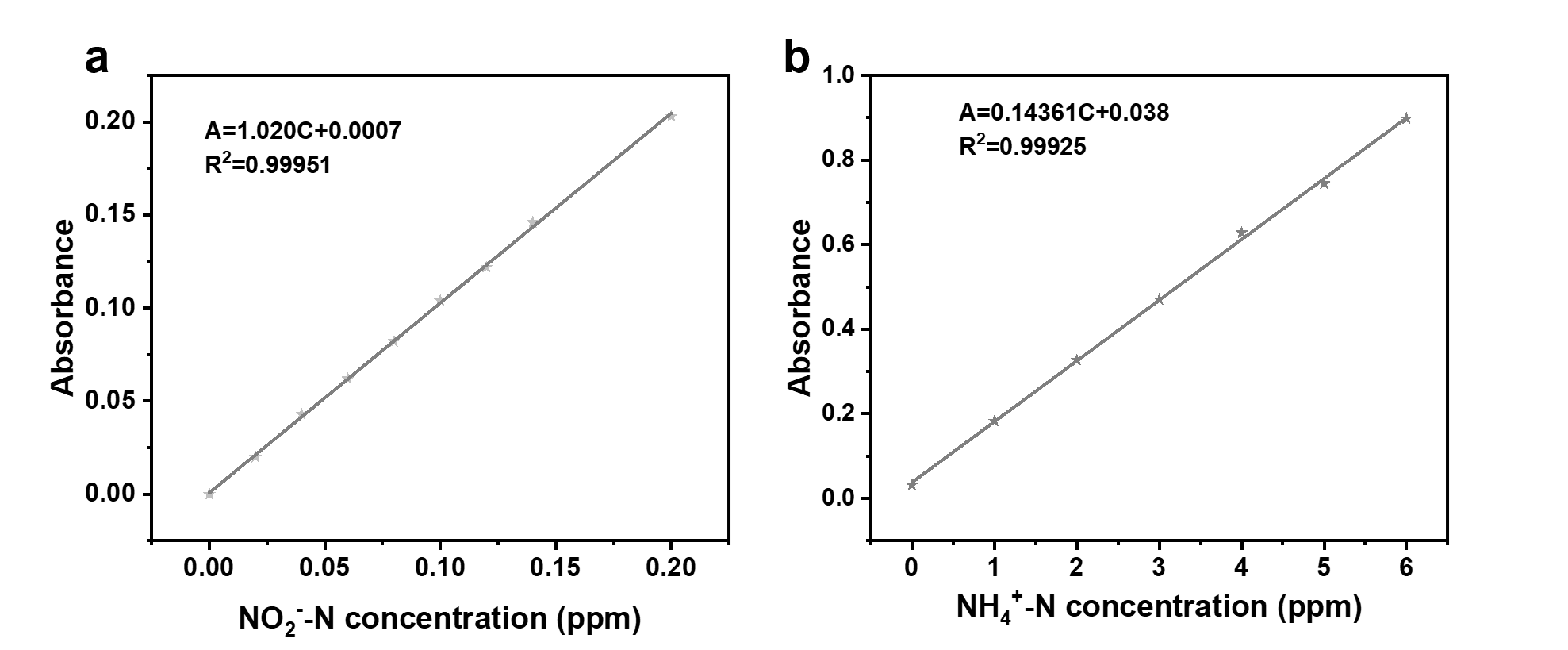


**Figure S13.** (a) The concentration-absorbance calibration curves of NO_2_^-^-N, (b) The concentration-absorbance calibration curves of NH_4_^+^-N.


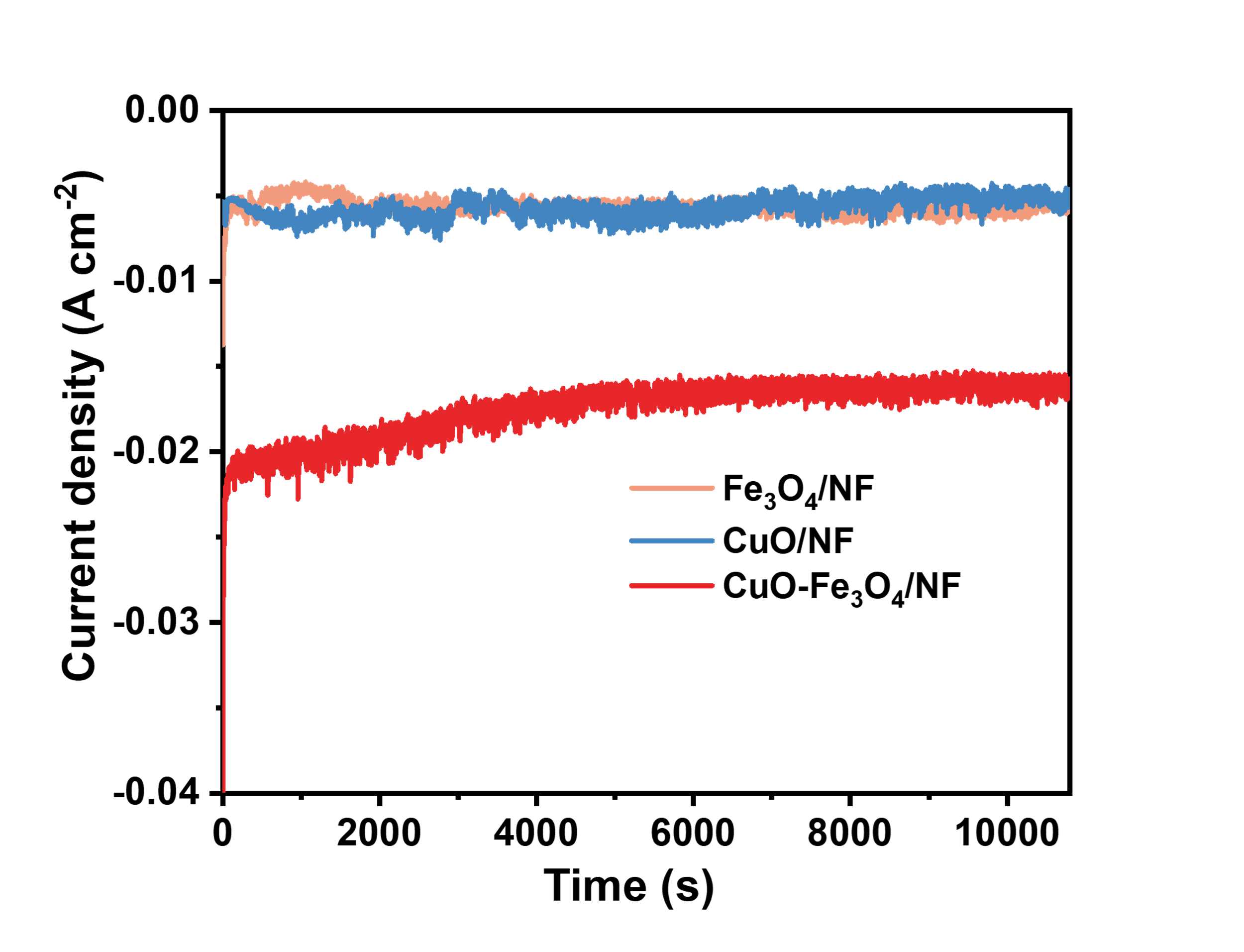


**Figure S14.** The corresponding chronoamperometry curves of CuO-Fe_3_O_4_/NF, CuO/NF, Fe_3_O_4_/NF.


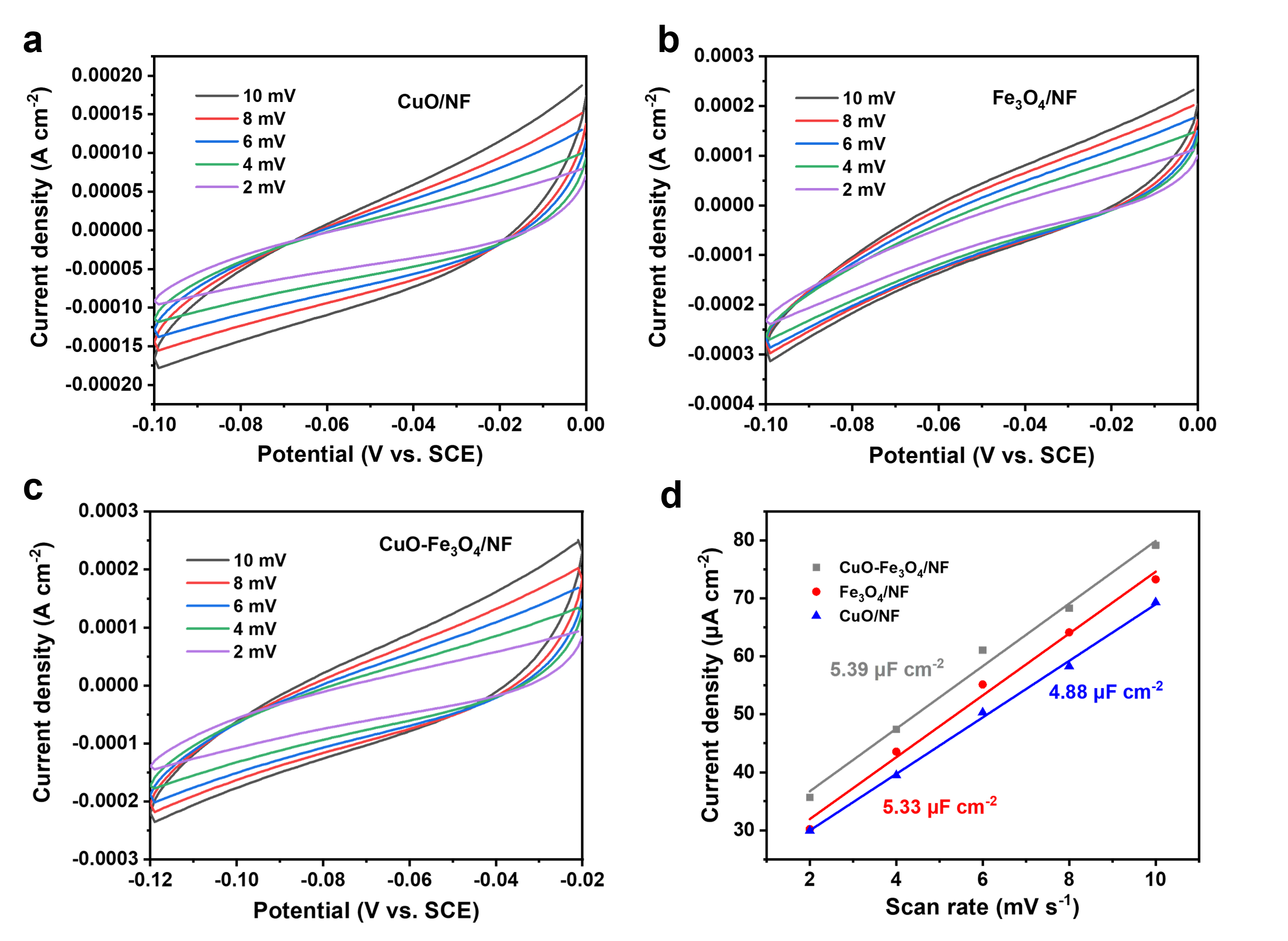


**Figure S15.** CV curves for (a) CuO/NF, (b) Fe_3_O_4_/NF and (c) CuO- Fe_3_O_4_/NF at different scan rates from 20 to 100 mV s-1, respectively. (d) Charging current density differences plotted against scan rates of CuO/NF, Fe_3_O_4_/NF and CuO-Fe_3_O_4_/NF.


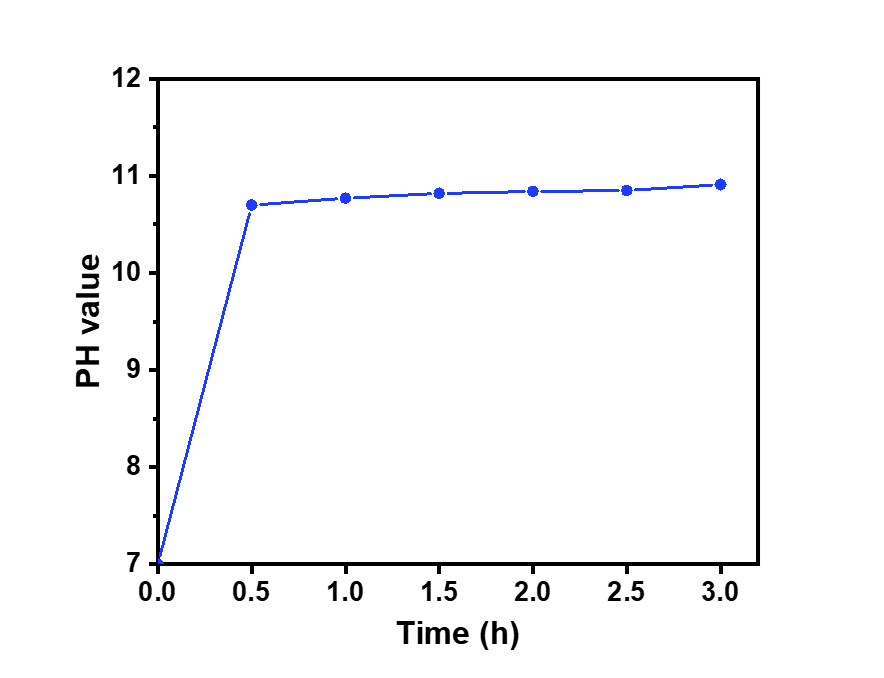


**Figure S16.** Changes in the pH of the electrolyte with the progress of the reaction.


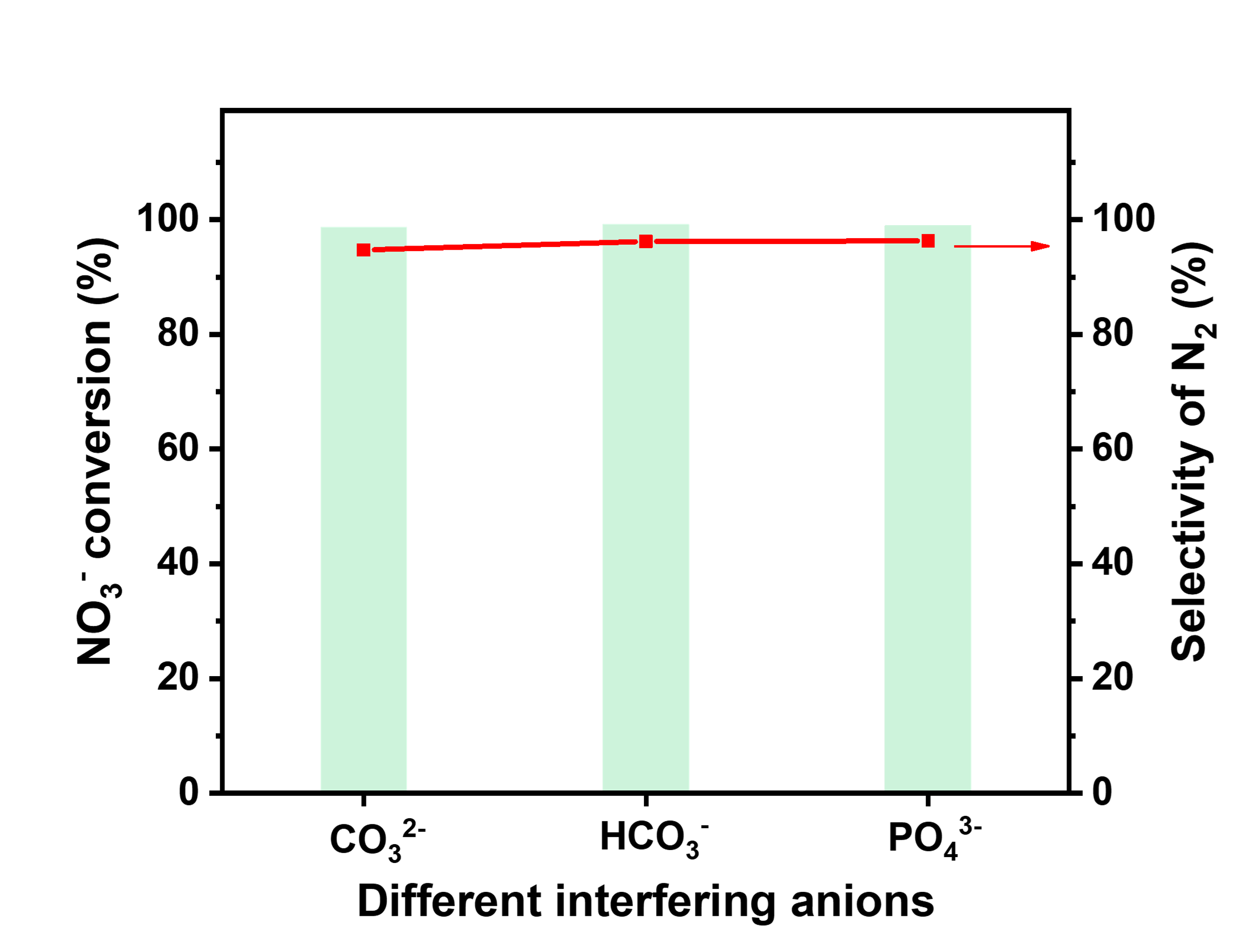


**Figure S17.** NO_3_RR activity over CuO-Fe_3_O_4_/NF with 100 ppm CO_3_^2^⁻, HCO_3_⁻ and PO_4_^3^⁻.


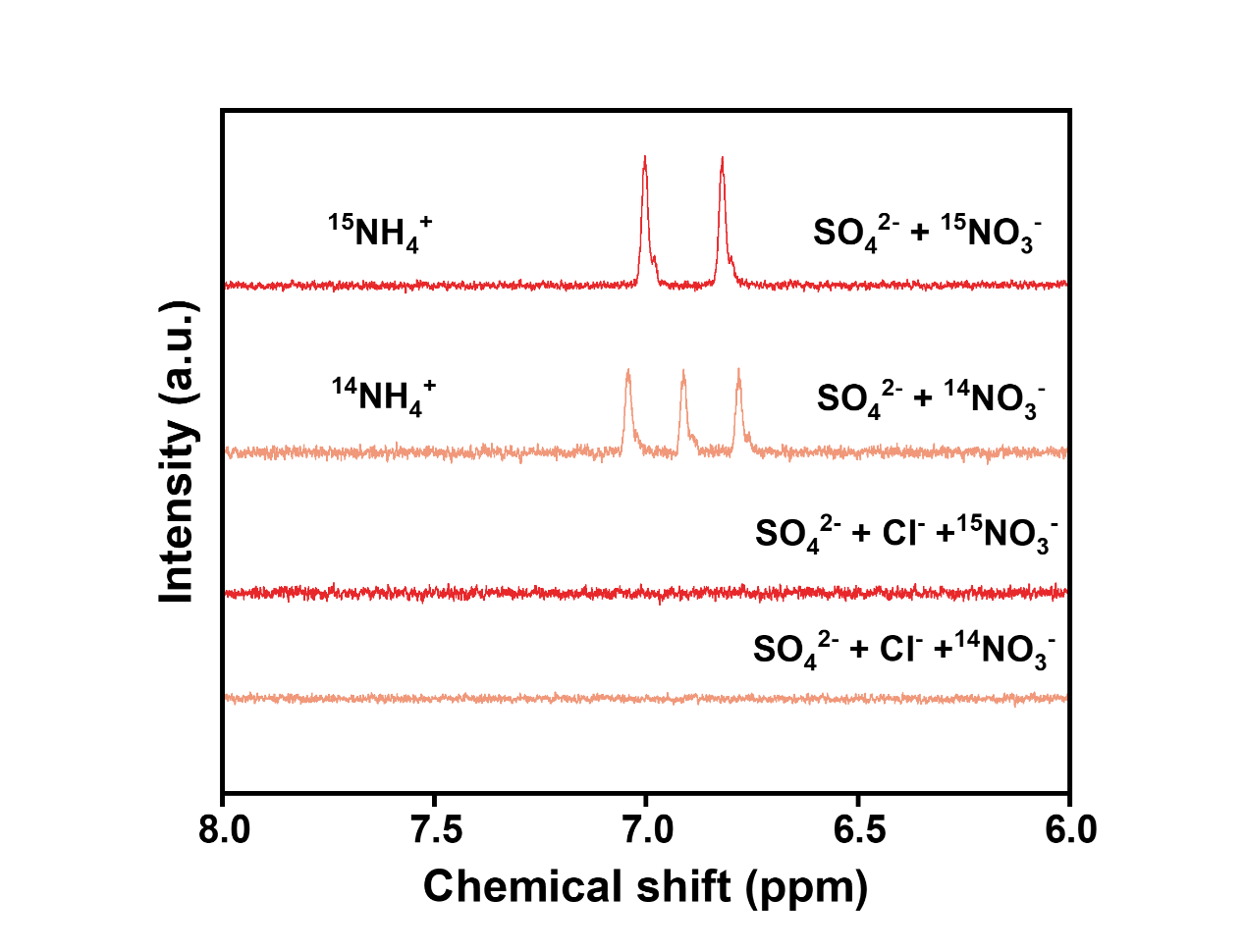


**Figure S18.** ^1^H NMR spectra of the generated ^14^NH_4_^+^ and ^15^NH_4_^+^.


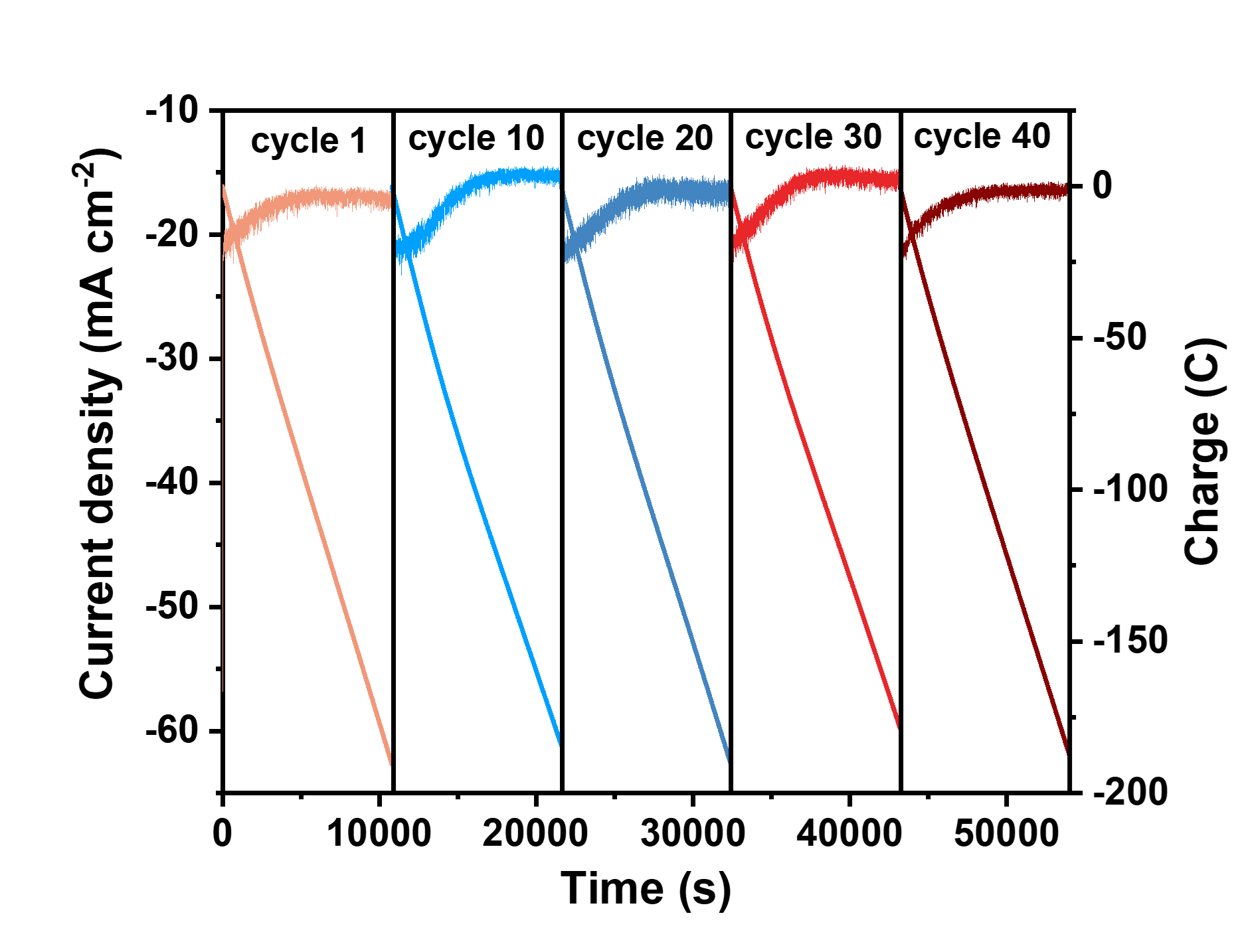

**Figure S19.** CA curves and the corresponding Coulomb consumptions, calculated using the integral area of CuO-Fe_3_O_4_/NFelectrode at the 1^st^, 10^th^, 20^th^, 30^th^, and 40^th^ cycles.


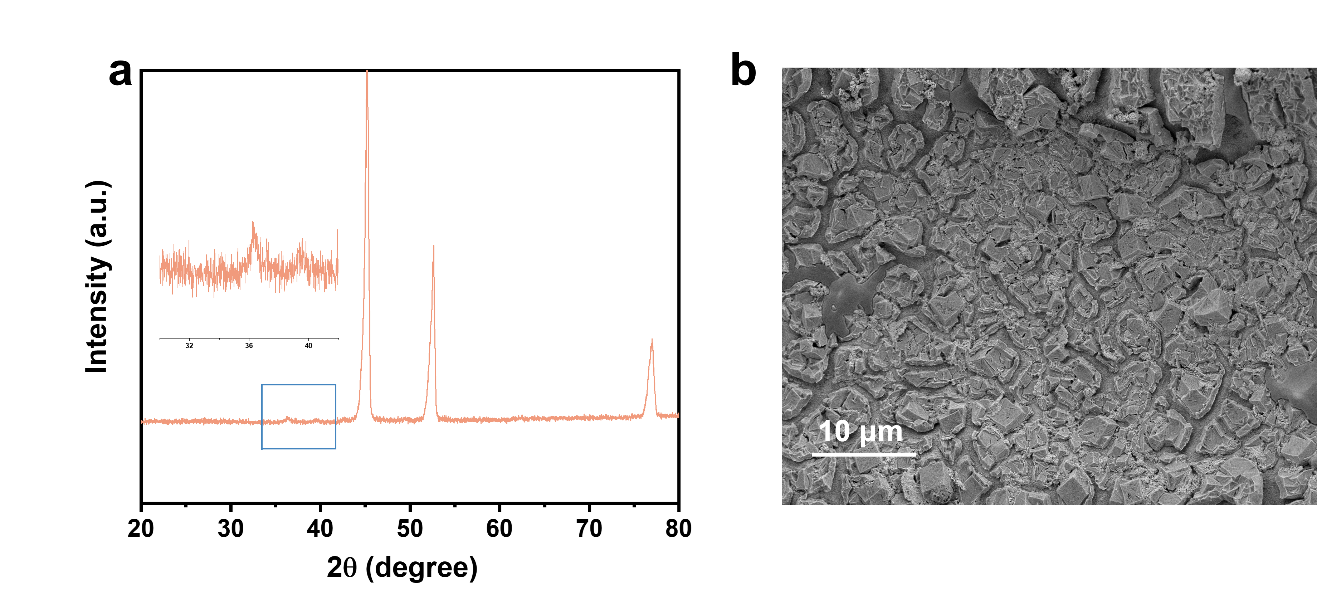


**Figure S20.** (a) XRD pattern of CuO-Fe_3_O_4_/NF after 40 cycles, (b) SEM image of CuO-Fe_3_O_4_/NF after 40 cycles.


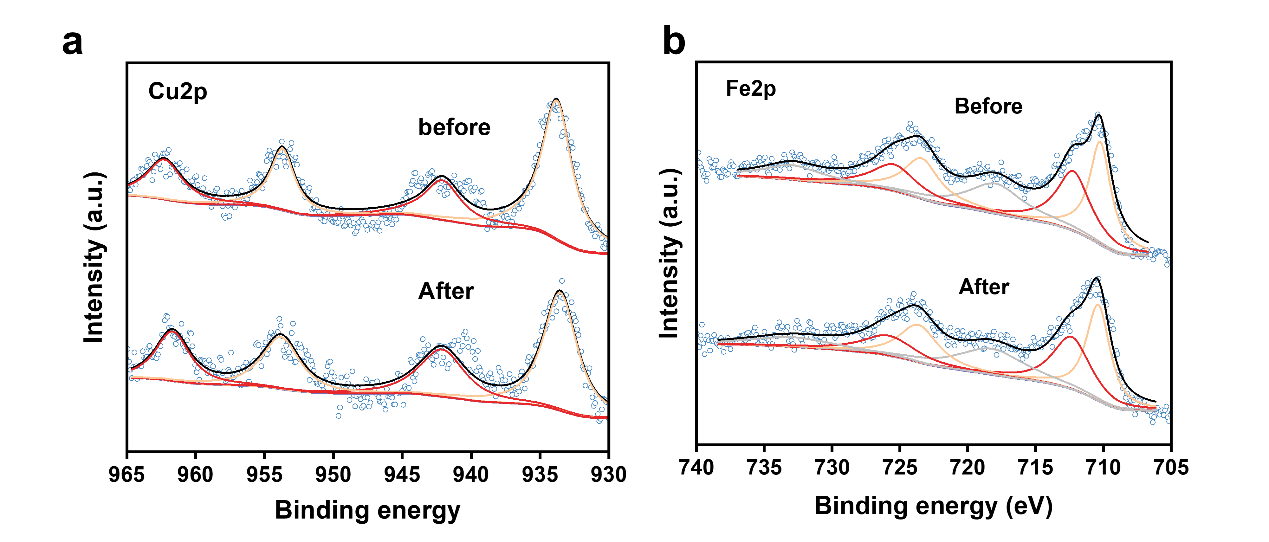


**Figure S21.** (a) Cu 2p XPS spectra of CuO-Fe_3_O_4_/NF before test and after 40 cycles, (b) Fe 2p XPS spectra of CuO-Fe_3_O_4_/NF before test and after 40 cycles.


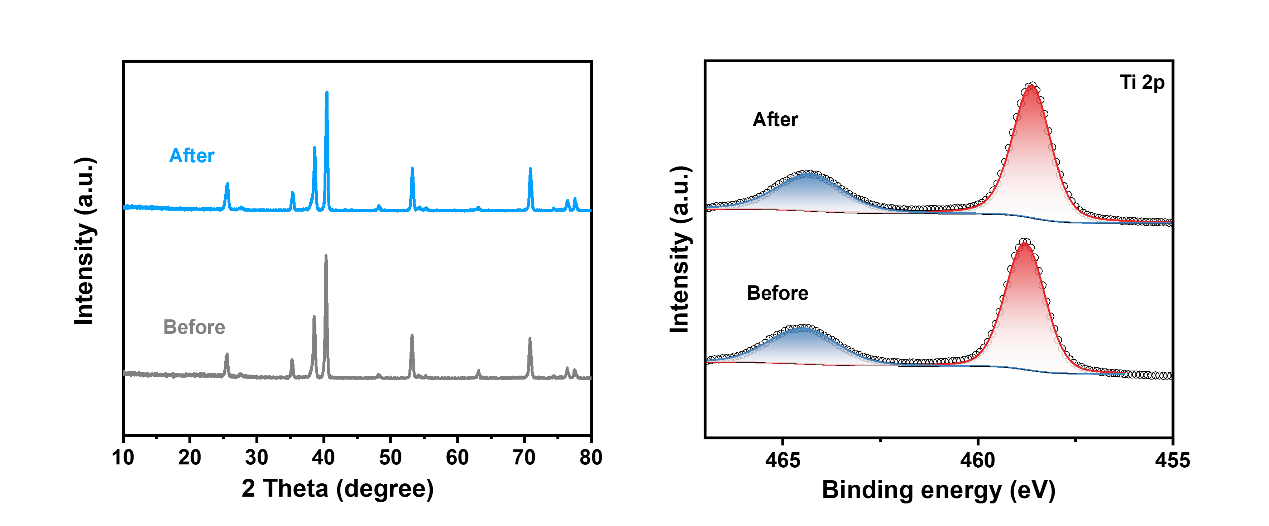


**Figure S22.** XRD pattern (left) and Ti 2p XPS spectra (right) of TiO_2_-NTAs photoanode before test and after 40 cycles.


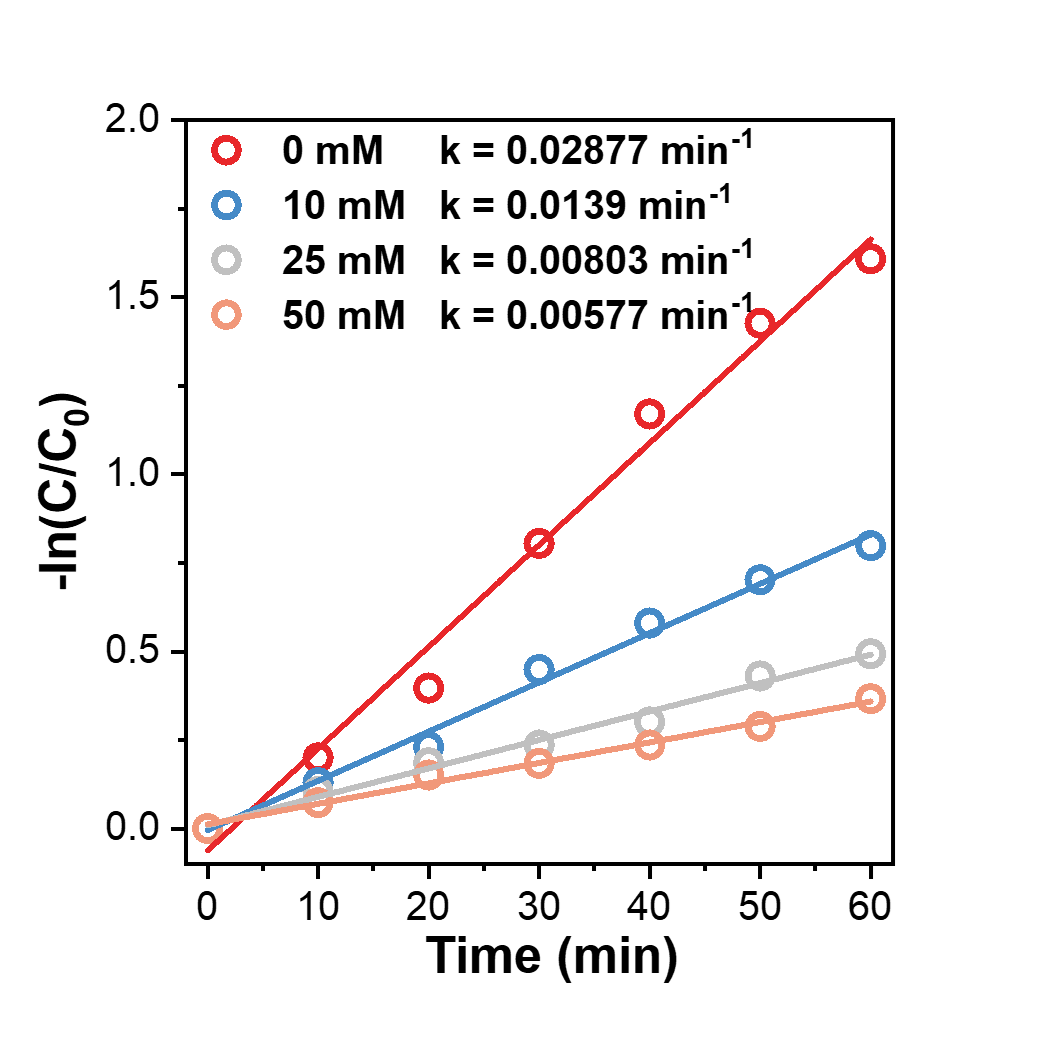


**Figure S23.** Linearized pseudo-first-order kinetics profiles of CuO-Fe_3_O_4_/NF with different TBA concentrations.


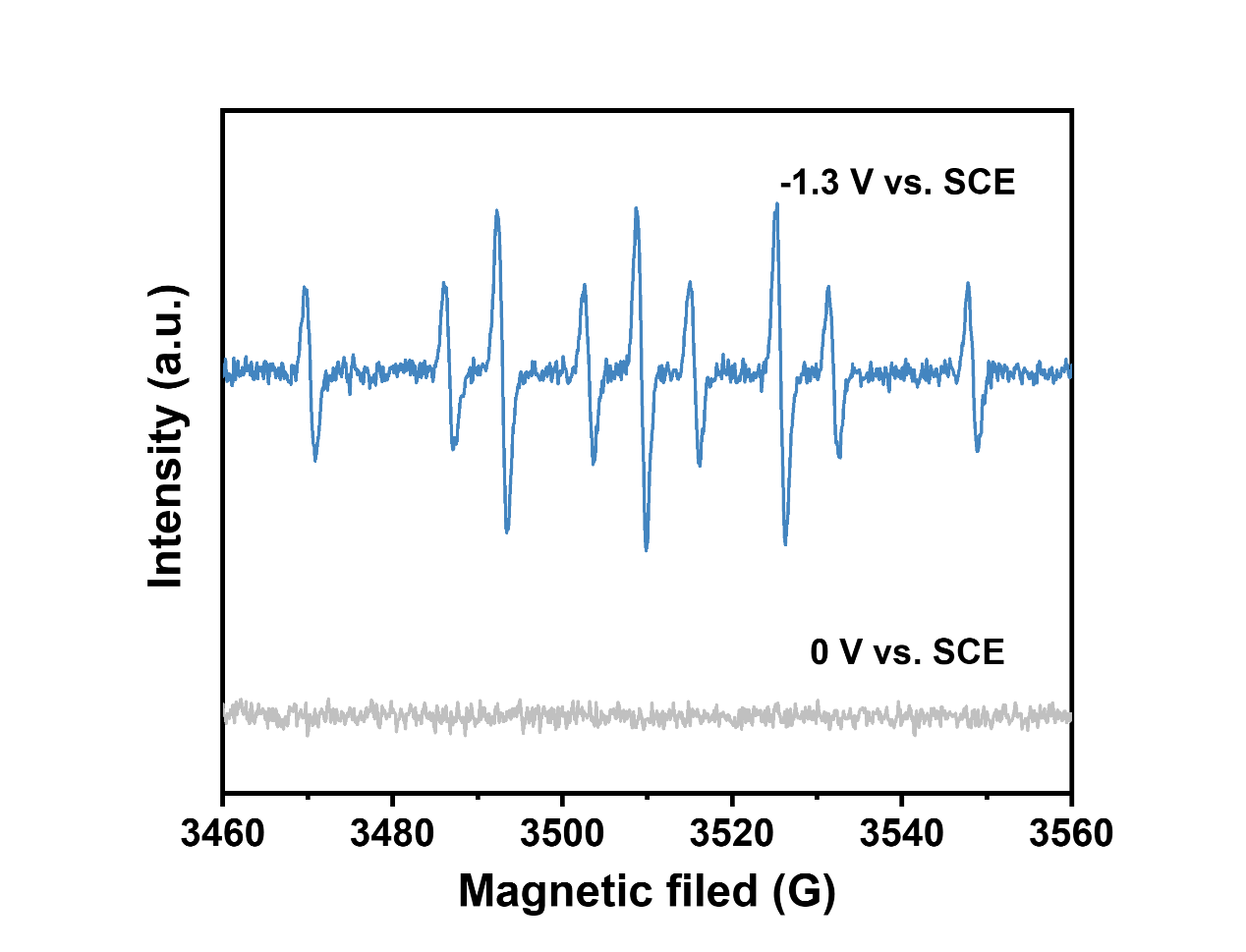


**Figure S24.** DMPO spin-trapping ESR spectra of CuO-Fe_3_O_4_/NF at different potential.


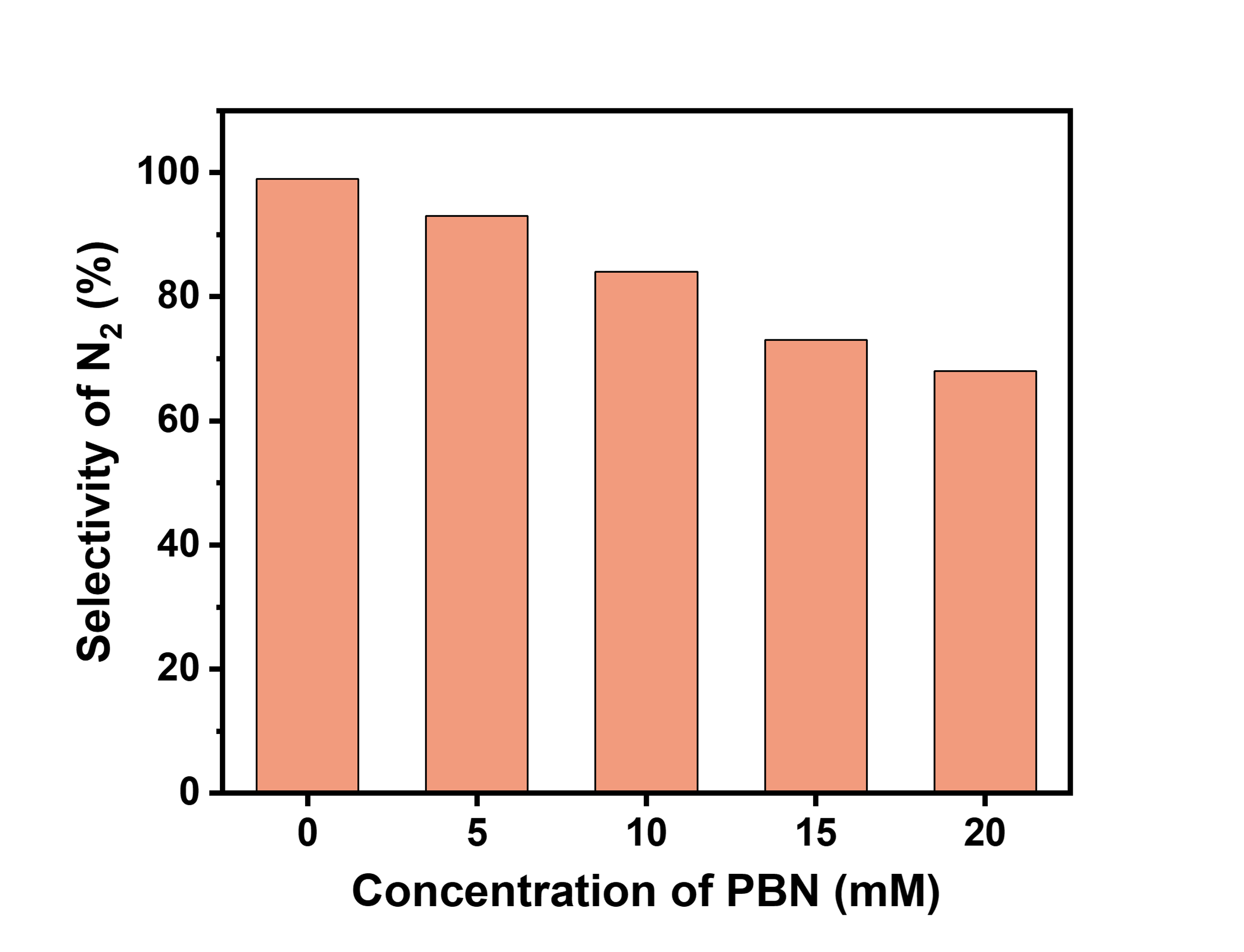


**Figure S25.**The nitrogen selectivity of CuO-Fe_3_O_4_/NF with different PBN concentrations at an applied potential of -1.3 V (vs. SCE) in 0.02 M Na_2_SO_4_ and 0.02 M NaCl electrolyte containing 100 mg/L NO_3_⁻ for 3 h.


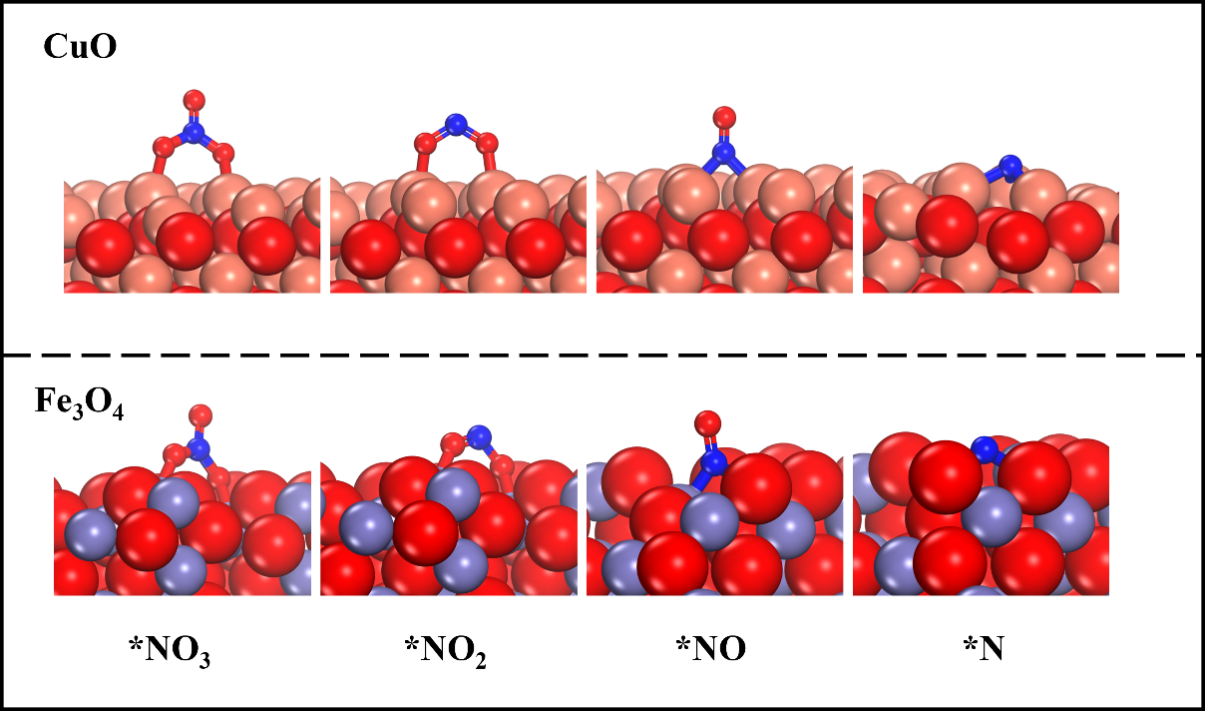


**Figure S26.** Structure models of key intermediates of NO_3_RR on CuO (0 0 2) and Fe_3_O_4_ (2 2 0) slabs. The red, blue, pink, and gray spheres represent O, N, Cu and Fe atoms, respectively.


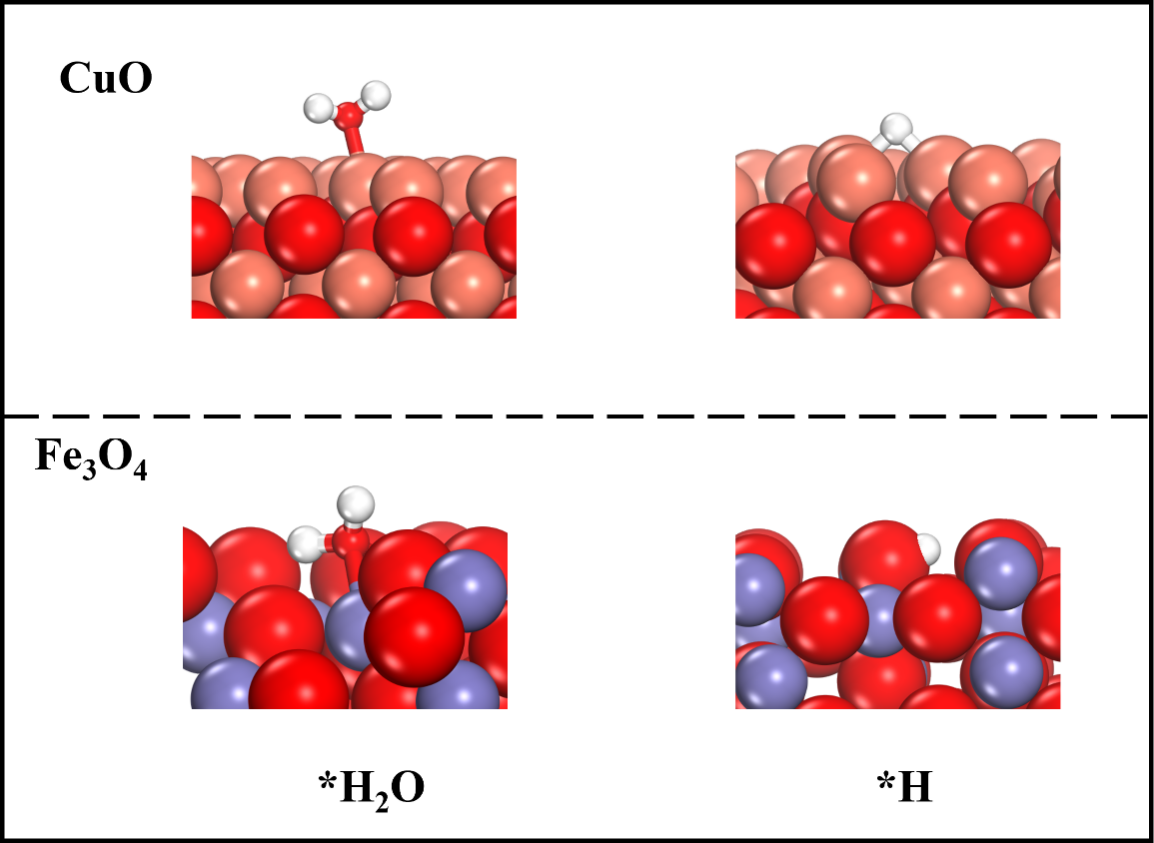


**Figure S27.** Structure models of H_2_O and H on CuO (0 0 2) and Fe_3_O_4_ (2 2 0) slabs. The red, white, pink, and gray spheres represent O, H, Cu and Fe atoms, respectively.


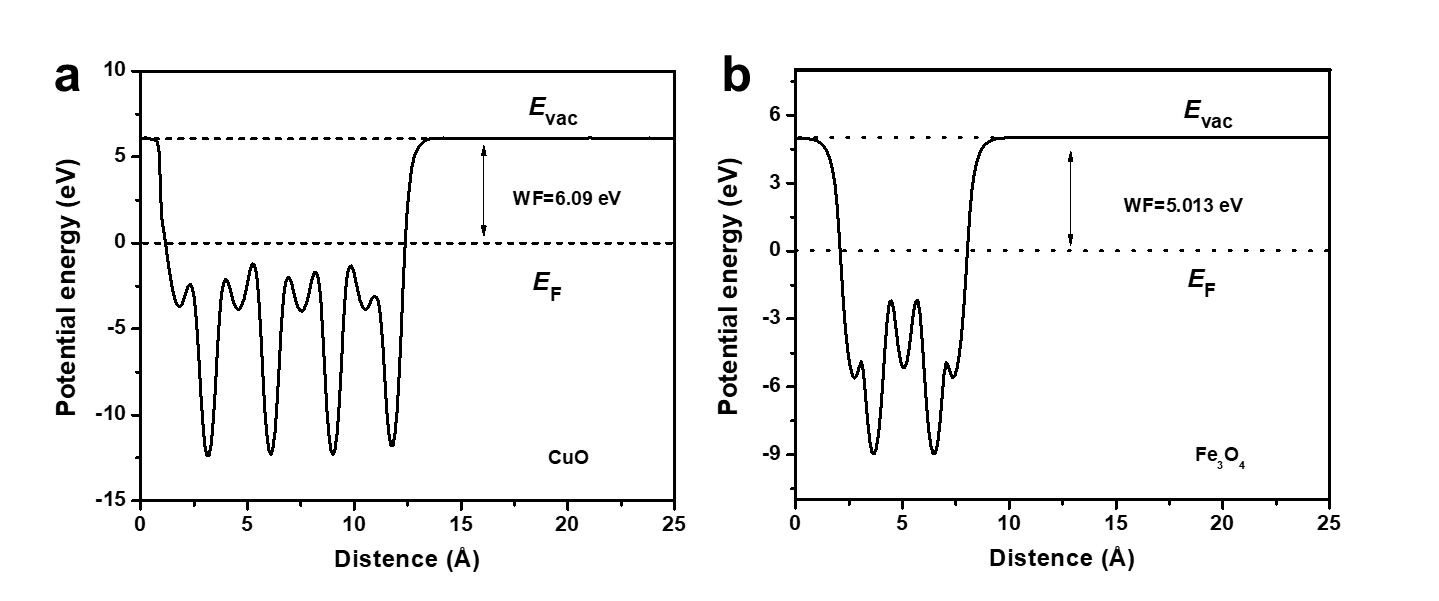


**Figure S28.**Work function of CuO a) and Fe_3_O_4_ b).


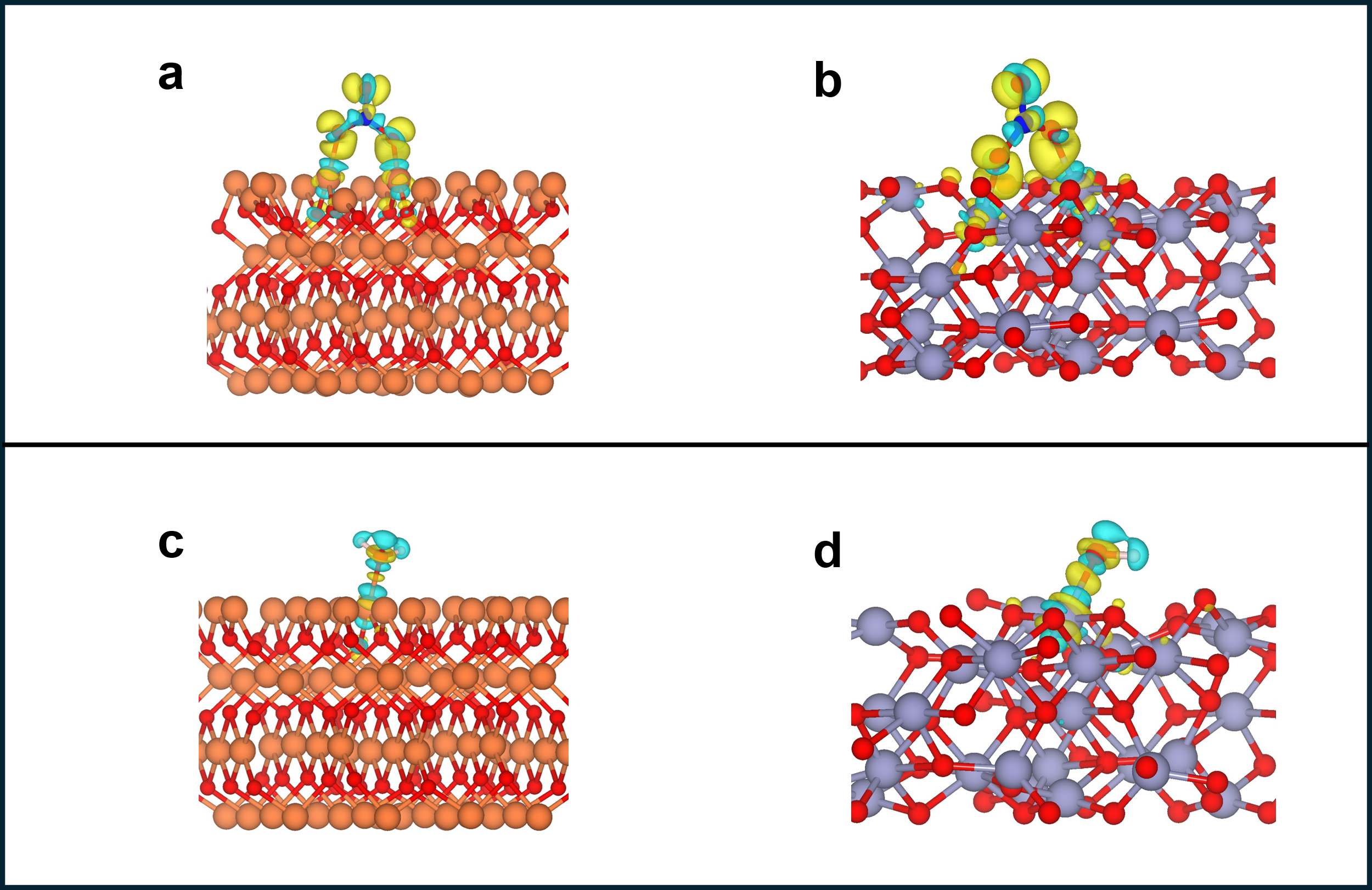


**Figure S29.**Charge density difference illustrating *NO_3_ a) and *H_2_O c) adsorption on CuO (0 0 2) surface. Charge density difference illustrating *NO_3_ b) and *H_2_O d) adsorption on Fe_3_O_4_ (2 2 0) surface.

**Table S1.** Electrocatalytic reduction performance of various catalysts.

| **Electrocatalyst** | **Electrolyte** | **Time** | **Cycles** | **Performance** | **Ref.** |
| --- | --- | --- | --- | --- | --- |
| RL-Fe_2_N@NC | 100 mg/L NO_3_^-^-N | 24 h | 40 | R(NO_3_^-^): 86%  S(N_2_): 97% | **^5^** |
| CL-Fe@C | 100 mg/L NO_3_^-^-N | 48 h | 5 | R(NO_3_^-^): 54%  S(N_2_): 98% | **^6^** |
| Fe@C-1 | 100 mg/L NO_3_^-^-N | 24 h | 5 | R(NO_3_^-^): 76%  S(N_2_): 98% | **^7^** |
| FeNi/g-mesoC/NF | 50 mg/L NO_3_^-^-N | 24 h | 30 | R(NO_3_^-^): 71%  S(N_2_): 25% | **^8^** |
| nZVI@OMC | 50 mg/L NO_3_^-^-N | 24 h | 6 | R(NO_3_^-^): 67%  S(N_2_): 74% | **^9^** |
| FeN-NC-140 | 40 mg/L NO_3_^-^-N | 24 h | 8 | R(NO_3_^-^): 90%  S(N_2_): 91% | **^10^** |
| Co-PBAs | 50 mg/L NO_3_^-^-N | 12 h | 20 | R(NO_3_^-^):100%  S(N_2_): 97% | **^11^** |
| Fe/Fe_3_C-NCNF | 100 mg/L NO_3_^-^-N | 24 h | 7 | R(NO_3_^-^): 75%  S(N_2_): 95% | **^12^** |
| B-Fe NCs | 100 mg/L NO_3_^-^-N | 24 h | 20 | R(NO_3_^-^): 80%  S(N_2_): 99% | **^13^** |
| Meso-Fe–N–C | 50 mg/L NO_3_^-^-N | 24 h | 6 | R(NO_3_^-^): 77%  S(N_2_): 85% | **^14^** |
| NiCu@N-C/NF | 7.14 mM NO_3_^-^ | 12 h | 10 | R(NO_3_^-^): 98%  S(N_2_): 99% | **^15^** |
| Co−CuOx | 20 N−mg L ^−1^ | 24 h | 10 | R(NO_3_^-^): 95%  S(N_2_): 96% | **^16^** |
| Fe#OMC | 400 mg/L NO_3_^-^-N | 24 h | 10 | R(NO_3_^-^): 92%  S(N_2_): 100% | **^17^** |
| Fe (II)-Fe (III) hydroxides | 100 mg/L NO_3_^-^-N | 12 h | 9 | R(NO_3_^-^): 96%  S(N_2_): 99% | **^18^** |
| PdCu/C | 300 mg/L NO_3_^-^-N | 24 h | 1 | R(NO_3_^-^): 56%  S(N_2_): 93% | **^19^** |
| CuO-Fe_3_O_4_/NF | 100 mg/L NO_3_^-^ | 3h | 40 | R(NO_3_^-^): 97%  S(N_2_): 99% | **This work** |

**Table S2.** The characteristics of natural water.

| **Indicators** | **value** |
| --- | --- |
| NO_3_⁻ | 1.5 mg L^-1^ |
| NO_2_⁻ | 0.34 mg L^-1^ |
| NH_4_^+^ | 0.02 mg L^-1^ |
| pH | 7.2 |

**References**

1. G. Kresse, J. Hafner, “Ab initio molecular dynamics for liquid metals.” *Phys. Rev. B* **1993**, *47* (1), 558.
2. G. Kresse, J. Furthmüller, “Efficiency of ab-initio total energy calculations for metals and semiconductors using a plane-wave basis set.” *Comput. Mater. Sci.* **1996**, *6* (1), 15-50.
3. J. Harl, L. Schimka, G. Kresse, “Assessing the quality of the random phase approximation for lattice constants and atomization energies of solids.” *Phys. Rev. B: Condens. Matter and Mater. Phys.* **2010**, *81* (11), 115126.
4. M. Bajdich, M. García-Mota, A. Vojvodic, J. Nørskov, A. Bell, “Theoretical investigation of the activity of cobalt oxides for the electrochemical oxidation of water.” *J. Am. Chem. Soc.* **2013**, *135* (36), 13521-13530.
5. H. Luo, S. Li, Z. Wu, Y. Liu, W. Luo, W. Li, D. Zhang, J. Chen, J. Yang, “Modulating the active hydrogen adsorption on Fe-N interface for boosted electrocatalytic nitrate reduction with ultra‐long stability.” *Adv. Mater.* **2023**, *35* (46), 2304695.
6. L. Su, D. Han, G. Zhu, H. Xu, W. Luo, L. Wang, W. Jiang, A. Dong, J. Yang, “Tailoring the assembly of iron nanoparticles in carbon microspheres toward high-performance electrocatalytic denitrification.” *Nano Lett.* **2019**, *19* (8), 5423-5430.
7. W. Hong, L. Su, J. Wang, M. Jiang, Y. Ma, J. Yang, “Boosting the electrocatalysis of nitrate to nitrogen with iron nanoparticles embedded in carbon microspheres.” *Chem. Commun.* **2020**, *56* (93), 14685-14688.
8. X. Chen, T. Zhang, M. Kan, D. Song, J. Jia, Y. Zhao, X. Qian, “Binderless and oxygen vacancies rich FeNi/graphitized mesoporous carbon/Ni foam for electrocatalytic reduction of nitrate.” *Environ. Sci. Technol.* **2020***,* *54* (20), 13344-13353.
9. W. Teng, N. Bai, Y. Liu, Y. Liu, J. Fan, W. Zhang, “Selective nitrate reduction to dinitrogen by electrocatalysis on nanoscale iron encapsulated in mesoporous carbon.” *Environ. Sci. Technol.* **2018**, *52* (1), 230-236.
10. J. Wang, L. Ling, Z. Deng, W. Zhang, “Nitrogen-doped iron for selective catalytic reduction of nitrate to dinitrogen.” *Sci. Bull.* **2020**, *65* (11), 926-933.
11. B. Xu, Z. Chen, G. Zhang, Y. Wang, “On-demand atomic hydrogen provision by exposing electron-rich cobalt sites in an open-framework structure toward superior electrocatalytic nitrate conversion to dinitrogen.” *Environ. Sci. Technol.* **2021**, *56* (1), 614-623.
12. J. Sun, W. Gao, H. Fei, G. Zhao, “Efficient and selective electrochemical reduction of nitrate to N_2_ by relay catalytic effects of Fe-Ni bimetallic sites on MOF-derived structure.” *Appl. Catal. B* **2022**, *301,* 120829.
13. Y. Lan, J. Chen, H. Zhang, W. Zhang, Y. Yang, “Fe/Fe_3_C nanoparticle-decorated N-doped carbon nanofibers for improving the nitrogen selectivity of electrocatalytic nitrate reduction.” *J. Mater. Chem. A* **2020**, *8* (31), 15853-15863.
14. F. Ni, Y. Ma, J. Chen, W. Luo, J. Yang, “Boron-iron nanochains for selective electrocatalytic reduction of nitrate.” *Chin. Chem. Lett.* **2021**, *32* (6), 2073-2078.
15. F. Fan, Y. Chen, X. Chen, Z. Wu, W. Teng, W. Zhang, “Atomically dispersed iron enables high-efficiency electrocatalytic conversion of nitrate to dinitrogen on a N-coordinated mesoporous carbon architecture.” *Appl. Catal. B* **2023**, *320*, 121983.
16. L. He, F. Yao, Y. Zhong, C. Tan, K. Hou, Z. Pi, S. Chen, X. Li, Q. Yang, “Achieving high-performance electrocatalytic reduction of nitrate by N-rich carbon-encapsulated Ni-Cu bimetallic nanoparticles supported nickel foam electrode.” *J. Hazard. Mater.* **2022***, 436*, 129253.
17. Y. Li, J. Ma, Z. Wu, Z. Wang, “Direct electron transfer coordinated by oxygen vacancies boosts selective nitrate reduction to N_2_ on a Co–CuO_X_ electroactive filter.” *Environ. Sci. Technol.* **2022**, *56* (12), 8673-8681.
18. X. Liu, Y. Wang, R. Jr, L Liu, X Qi, “Synthesis of self-renewing Fe (0)-dispersed ordered mesoporous carbon for electrocatalytic reduction of nitrates to nitrogen.” *Sci. Total Environ.* **2022***, 836*, 155640.
19. R. Mao, H. Zhu, K. Wang, X. Zhao, “Selective conversion of nitrate to nitrogen gas by enhanced electrochemical process assisted by reductive Fe (II)-Fe (III) hydroxides at cathode surface.” *Appl. Catal. B* **2021**, *298*, 120552.
